# Establishment of a Molecular Serotyping Scheme and a Multiplexed Luminex-Based Array for *Enterobacter aerogenes*

**DOI:** 10.3389/fmicb.2018.00501

**Published:** 2018-03-19

**Authors:** Xi Guo, Min Wang, Lu Wang, Yao Wang, Tingting Chen, Pan Wu, Min Chen, Bin Liu, Lu Feng

**Affiliations:** ^1^Key Laboratory of Molecular Microbiology and Technology, Ministry of Education–Tianjin Economic-Technological Development Area, Tianjin, China; ^2^Tianjin Key Laboratory of Microbial Functional Genomics, Tianjin Economic-Technological Development Area, Tianjin, China; ^3^TEDA Institute of Biological Sciences and Biotechnology, Nankai University–Tianjin Economic-Technological Development Area, Tianjin, China; ^4^Shanghai Municipal Center for Disease Control and Prevention, Shanghai, China

**Keywords:** *Enterobacter aerogenes*, polysaccharide gene cluster, molecular serotyping system, antigenic scheme, Luminex-based array

## Abstract

Serotyping based on surface polysaccharide antigens is important for the clinical detection and epidemiological surveillance of pathogens. Polysaccharide gene clusters (PSgcs) are typically responsible for the diversity of bacterial surface polysaccharides. Through whole-genome sequencing and analysis, eight putative PSgc types were identified in 23 *Enterobacter aerogenes* strains from several geographic areas, allowing us to present the first molecular serotyping system for *E. aerogenes*. A conventional antigenic scheme was also established and correlated well with the molecular serotyping system that was based on PSgc genetic variation, indicating that PSgc-based molecular typing and immunological serology provide equally valid results. Further, a multiplex Luminex-based array was developed, and a double-blind test was conducted with 97 clinical specimens from Shanghai, China, to validate our array. The results of these analyses indicated that strains containing PSgc4 and PSgc7 comprised the predominant groups. We then examined 86 publicly available *E. aerogenes* strain genomes and identified an additional seven novel PSgc types, with PSgc10 being the most abundant type. In total, our study identified 15 PSgc types in *E. aerogenes*, providing the basis for a molecular serotyping scheme. From these results, differing epidemic patterns were identified between strains that were predominant in different regions. Our study highlights the feasibility and reliability of a serotyping system based on PSgc diversity, and for the first time, presents a molecular serotyping system, as well as an antigenic scheme for *E. aerogenes*, providing the basis for molecular diagnostics and epidemiological surveillance of this important emerging pathogen.

## Introduction

*Enterobacter aerogenes* is a Gram-negative bacterium that is ubiquitous in the human gastrointestinal tract and various other environments and is generally non-pathogenic to healthy humans (Chen et al., 2015). However, *E. aerogenes* has become an important opportunistic pathogen since the early 1990s and has frequently been isolated from respiratory, urinary, blood, and gastrointestinal tract infections (Langley et al., 2001). Moreover, *E. aerogenes* has been reported to readily cause septic shock in patients, thus leading to high mortality rates (Lavigne et al., 2012). *E. aerogenes* strains that are isolated from hospitalized patients generally exhibit high resistance to commonly used broad-spectrum antibiotics (De Gheldre et al., 1997; Chen et al., 2008; Lavigne et al., 2013). Consequently, this species has been considered an important emerging multidrug resistant (MDR) pathogen over the past two decades (Chevalier et al., 2008).

Surface polysaccharides, particularly the heat-stable somatic (O) and/or capsular (K) antigens, are major targets for both host immune systems and bacteriophages and therefore are some of the most variable constituents of the cell. The genes involved in surface polysaccharide synthesis are generally arranged in a gene cluster, and most of the variation among polysaccharides is due to genetic variation in the polysaccharide gene clusters (PSgcs), thereby providing the molecular basis for serotyping. These genes are commonly classified into three main classes: (i) nucleotide sugar precursor synthesis genes for sugars that are specific to the particular polysaccharide; (ii) sugar transferase genes that are associated with the O- or K-unit assemblies, and which are specific for the donor and acceptor sugars and generate a specific linkage between them; and (iii) genes (*wzx/wzy* or *wzm/wzt*) for O- or K-unit translocation and polymerization (Liu et al., 2014). In addition to the three gene classes above, a *wzi-wza-wab-wzc* gene set that is highly conserved among different serotypes exists in the locus of most *Klebsiella* capsular types and the group 1 capsule of *E. coli* (Whitfield, 2006; Pan et al., 2015). The protein complex Wza/b/c accepts the polymer made by Wzy and transports it to the cell surface, forming the capsule (K) antigen (Whitfield, 2006). The remainder, Wzi, is proposed to play a role in capsule assembly, but the mechanistic details of this process remain unclear (Rahn et al., 2003). The complete O-antigen is ligated to the lipid A/core by WaaL, which is encoded by the *waaL* gene in the core gene cluster, thus forming the LPS structure. The K-repeat polymer can also be added to the lipid A/core, and this is known as the K_LPS_, which does not require the Wza/b/c complex (Hu et al., 2013).

The recent development of molecular detection methods, including pulsed-field gel electrophoresis (PFGE), multilocus sequence typing (MLST), plasmid fingerprinting (PF), restriction fragment length polymorphism (RFLP) and gene-specific PCR, have advanced the ability to identify pathogens. Ribotyping, arbitrarily primed PCR and PFGE assays have been reported to be used for epidemiological study of nosocomial *E. aerogenes* isolates (Grattard et al., 1995; Jalaluddin et al., 1998). However, serotyping based on the diversity of surface polysaccharides that underlie antigenic schemes remains the ‘gold standard’ for detecting pathogenic strains in clinical specimens and environmental samples, as well as for epidemiological surveillance and tracing (Wang et al., 2010). In contrast to widely studied pathogens, including *Salmonella* spp. (Guibourdenche et al., 2010), *E. coli* (Orskov et al., 1977), *S. pneumoniae* (Konradsen and Pneumococcus Reference laboratories in Europe, 2005), and *Vibrio parahaemolyticus* (Chen et al., 2012) and other major pathogens, antigenic schemes for some important emerging pathogens have not been established. Furthermore, even the genetic and structural basis for serological diversity has not yet been elucidated in several of these emerging pathogens. The development of next-generation sequencing (NGS) has made it possible to rapidly detect almost all genetic features of numerous pathogens, including elucidation of putative PSgcs in novel, emerging pathogenic bacteria. Consequently, a molecular serotyping scheme could be developed based on the sequence diversity of PSgcs that is theoretically consistent with the traditional antigenic system based on the structural diversity of surface polysaccharide antigens.

Here, the genomes of 23 *E. aerogenes* strains were sequenced, and eight forms of putative PSgcs (1–8) were obtained and analyzed, providing confirmation of the molecular serotyping scheme for *E. aerogenes*. Antisera against type strains that represented each PSgc type were then produced and showed good absorption specificity to their homologous strains, indicating a high level of accuracy for the molecular serotyping scheme that we presented. Furthermore, a Luminex bead-based suspension array targeting these eight PSgc types was developed and evaluated for specificity and sensitivity in double-blind tests. We then examined another 86 *E. aerogenes* strains with genome sequences available in the GenBank database and identified an additional seven new gene cluster types (PSgcs 9–15).

## Materials and Methods

### Sequencing and Bioinformatic Analyses

Bacterial cultivation and DNA extraction were conducted in a BS-2 lab.

Whole-genome sequencing of 23 *E. aerogenes* strains (Supplementary Table 1) was performed on the Solexa paired-end sequencing platform. Genomic DNA was sheared, polished, and prepared using the Illumina Sample Preparation Kit according to the manufacturer’s protocols. Genomic libraries were constructed that contained 500-bp paired-end inserts, and sequencing was performed with Solexa sequencing technologies (Illumina, Inc.) to produce ∼100-fold coverage for each genome. Sequence reads were assembled using the *de novo* genome-assembly program Velvet to generate multi-contig draft genomes. Gaps within the putative PSgc were closed by directed PCR, and the products were sequenced using BigDye terminator chemistry on ABI 3730 capillary sequencers.

Artemis (Rutherford et al., 2000) was used to annotate genes, and the lockMaker program (Henikoff et al., 1995) was used to identify conserved motifs. BLAST and PSI-BLAST (Altschul et al., 1997) were used to search genes and proteins against available databases, including GenBank^1^ and the Pfam protein motif databases^2^. The TMHMM analysis program v2.0^3^ was used to identify potential transmembrane domains within protein sequences.

### Identification of Putative PSgcs From Genomes

Eighty-six *E. aerogenes* genome sequences were downloaded from the GenBank database (Supplementary Table 1) and combined with the 23 genome sequences obtained in this study to assess PSgc distributions. Putative PSgc sequences that were located between the two housekeeping genes *galF* and *gnd* were retrieved from the genomes. PSgc sequences sharing high DNA or protein level identity (>97%) and possessing the same gene organization were considered identical serotypes.

### Preparation of Antigens for Immunization

All strains that were used for immunization were cultivated in 400 ml of media and were harvested by centrifugation at 5,000 × *g* for 20 min, washed in 20 ml of 0.85% NaCl, and suspended in 20 ml of 0.85% NaCl (approximately 10^11^ cells/ml). The cell suspension was subsequently treated with 0.5% formaldehyde overnight. Only strains that did not exhibit autoagglutination were used for immunization. The resultant antigens were stored at 4°C.

### Production of Antisera

Adult New Zealand white rabbits (2.5 kg weight) were injected intravenously with the prepared antigens. Three rabbits were used for each *Enterobacter* strain. At 3-day intervals, injections were given at doses of 0.5, 1, 2, and 4 ml. Rabbits were exsanguinated 8 days after the final injection, and the separated antisera were stored at 4°C.

### Tube Agglutination

Serotyping and titer determination were performed by tube agglutination. For antigen preparation, cultures were taken from agar plates after incubation at 37°C overnight, autoclaved, and then washed in 20 ml of 0.85% NaCl, and suspended in 20 ml of 0.85% NaCl. Tube agglutination was performed in 96-well U-bottom microtiter plates with 25 μl of antigen and 25 μl of twofold diluted antiserum in PBS (50 mM phosphate buffer, 150 mM NaCl, pH 7.2). Microtiter plates were incubated at 37°C for 4 h and then stored at 4°C overnight. The titer was determined based on the most diluted concentration of a serum that gave a positive reaction compared to the PBS negative control.

### Antiserum Absorption

To assess the absorption of agglutinins, cell suspensions were prepared by inoculating moist, thickly poured (∼1 ml medium per plate) 90-mm infusion agar plates. Plates were incubated in an upright position for 18–24 h at 37°C. Cultures from the plates were then suspended in 0.85% NaCl and treated with 0.5% formaldehyde overnight and then centrifuged at 5,000 × *g* for 20 min. The cell pellets were then gently washed with 0.85% NaCl three times and resuspended in 2 ml of antisera. The mixtures were incubated at room temperature for 2 h and subsequently centrifuged at 10,000 × *g* for 15 min, and the supernatants were collected. The absorbed antisera were then tested against all antigens that reacted with the unabsorbed antisera. This process was repeated until cross-reactions no longer occurred.

### Design of Primers and Probes

PSgc1-8 specific primers and probes were designed based on the DNA sequences that were obtained in this study using Primer Premier v5.0 software (Premier Biosoft International, Palo Alto, CA, United States). The specificity of each individual primer pair was confirmed using BLAST program and was subsequently validated by a single PCR amplification. For Luminex-based detection, each probe was synthesized with a 5′ amino modifier C12 that enabled coupling to the carboxyl group located on the microsphere, and each reverse primer was labeled with biotin at its 5′ end.

### Multiplex PCR Amplification

Multiplex PCR amplification was performed in a 50 μl reaction mixture consisting of 100 ng of genomic DNA, 1× Goldstar PCR buffer, 20 μM of each deoxynucleoside triphosphate (dNTP), 2.5 units of Goldstar DNA polymerase, 0.5 μM of each forward primer, and 2 μM of each reverse primer. The reaction parameters were as follows: hot start at 95°C for 5 min; amplification stage of 30 cycles at 95°C for 30 s, 60°C for 30 s, and 72°C for 1 min, followed by a final extension at 72°C for 5 min.

### Hybridization and Luminex Analysis

Each PSgc probe was bound to a different carboxylated microsphere as described previously (Chen et al., 2010). The working microspheres consisted of eight types of beads, and each bead was coupled to a PSgc-specific probe. Hybridization was performed in a 50 μl mixture that included 17 μl of biotin-labeled PCR product and 33 μl of working microspheres, with denaturation at 95°C for 10 min and incubation at 55°C for 17 min in a thermal cycler. The hybridization product was then transferred to a filter plate and washed three times with 1× TMAC buffer at 1,000 rpm for 1 min. For detection, 80 μl of streptavidin-R-phycoerythrin in 1× TMAC buffer was added to each well, followed by incubation at 53°C for 20 min. Finally, the signals for each set of beads were measured using a Bio-plex 100 reader (Bio-Rad) according to the manufacturer’s protocol. Data were analyzed using Bio-plex Manager 4.0, and the data are presented as the median fluorescence intensity (MFI). The cut-off value for a positive result was defined as three times greater than the mean MFI value of the background.

### Ethics Statement

All animal experiments were performed in accordance with the standards established in the Guide for the Care and Use of Laboratory Animals published by the Institute of Laboratory Animal Resources of the National Research Council (United States). The animal research procedures were approved by the Institutional Animal Care Committee at Nankai University and Tianjin Institute of Pharmaceutical Research New Drug Evaluation Co., Ltd. (IACUC number: 2016032102), Tianjin, China. Efforts were made to minimize animal suffering and to reduce the number of animals used.

## Results

### General Features of Putative PSgcs in *E. aerogenes*

In this study, genomes were sequenced from two reference *E. aerogenes* strains from the American Type Culture Collection (ATCC), two from the German Collection of Microorganisms and Cultures (DSMZ), and 19 clinical strains that were isolated from the Shanghai Municipal Center for Disease Control and Prevention (SCDC) during 2012–2015 (Supplementary Table 1). Putative PSgc regions of *E. aerogenes* were first located by us, and a total of eight different putative PSgc types were found. All eight putative PSgcs mapped between two housekeeping genes, *galF* and *gnd*, and ranged in size from 11,194 bp to 23,479 bp, with all genes transcribed from *galF* and *gnd*. The average % GC content of all putative PSgcs is ∼40%, which is significantly lower than for the *E. aerogenes* genome as a whole (55%). The PSgc allocation and accession numbers for all type strains are summarized in Supplementary Table 2.

**FIGURE 1:**
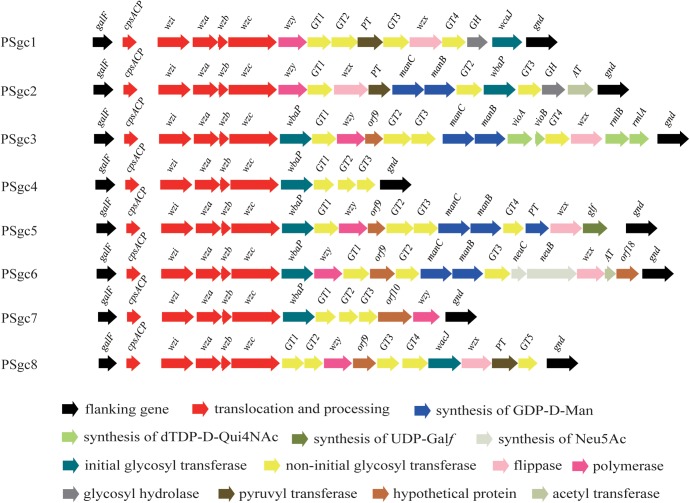
Schematic diagram of the eight putative PSgc types identified from the 23 *Enterobacter aerogenes* strain genomes sequenced in this study. Genes are represented by arrows and colored according to the gene key at the bottom with gene names indicated above each arrow.

In each PSgc, the conserved gene set *cpsACP-wzi-wza-wzb-wzc* was located at the 5′ end. Further, an initial glycosyltransferase gene (*wbaP* or *wcaJ*) was present, whose products have been characterized as initial glycosyl transferases (ITs) that transfer galactose-1-phosphate or glucose-1-phosphate, respectively, to undecaprenol phosphate to initiate polysaccharide synthesis (Liu et al., 1993; Patel et al., 2012). In several PSgc types, *manCB*, which is responsible for the formation of GDP-D-Man (Liu et al., 2008; Perepelov et al., 2015), *rmlAB/vioAB* for dDTP-D-Qui4NAc (Wang et al., 2007), and *glf* for UDP-Gal*f* (Nassau et al., 1996), were also presented. Biosynthetic pathways for all putative rare sugars present in *E. aerogenes* surface polysaccharides are indicated in Supplementary Figure 1. Meanwhile, each PSgc possessed non-initial glycosyltransferase genes (GTs), and oligosaccharide unit processing genes were observed in most PSgc types (**Figure 1**). Characteristics of open reading frames (ORFs) for all putative PSgc types are summarized in Supplementary Table 3.

A conserved 39 bp JUMPstar sequence has been reported to be present in the non-coding regions upstream of PSgcs in several bacteria, and it was proposed to play two possible roles, regulating the transcription of PSgcs and involvement in the recombination of sugar pathway and assembly genes between loci (Hu et al., 2013). We aligned the non-coding regions of each PSgc between *cpsACP* and *wzi* using MEGA 4 software and identified homologous JUMPstar sequences that were also present in these gene clusters (Supplementary Figure 2).

The results outlined above indicate that the genetic region between *galF* and *gnd* should be a PSgc, and a potential molecular serotyping system could be presented based on the genetic diversity of PSgc types.

### The Conventional Antigenic Scheme Correlated Well With the Molecular Serotyping System Based on PSgc Genetic Variation

Antisera were prepared using each type strain that represented PSgc types 1–8 and tested against all isolates. The homologous and heterologous titers of these antisera are summarized in **Table 1**. Generally, homologous titers were high, varying from 320 to 1280. However, none of the antisera were specific for only their homologous strains, with the exception of PSgc6 antiserum, and each antiserum resulted in unique cross-agglutination with one or more of the other antigens. Thus, the specific antisera needed to be absorbed with the corresponding heterologous antigens. Each antiserum agglutinated with only the corresponding homologous antigen after absorption, with serum titers ranging from 160 to 640 (**Table 2**). The eight absorbed antisera were then tested against all available strains, and each produced specific agglutination only against the strains that carried the same PSgc. These results suggested that the traditional immunoassay system was perfectly consistent with the molecular serotyping scheme that we developed based on PSgc diversity.

**Table 1:** Agglutination of absorbed *E. aerogenes* antisera.

		Agglutinin titers to each PSgc type strain							
	
Antiserum	G2351 (PSgc1)	G5305 (PSgc2)	G5306 (PSgc3)	G5307 (PSgc4)	G5308 (PSgc5)	G5310 (PSgc6)	G5313 (PSgc7)	G5319 (PSgc8)
PSgc1	1280	-	80	-	-	-	80	-
PSgc2	-	320	-	40	-	-	-	-
PSgc3	-	-	640	-	-	-	40	-
PSgc4	-	160	-	1,280	-	-	40	40
PSgc5	-	-	20	-	320	-	-	-
PSgc6	-	-	-	-	-	320	-	-
PSgc7	40	-	40	-	-	-	640	40
PSgc8	-	-	40	20	-	-	-	640


**Table 2:** Agglutination of absorbed *E. aerogenes* antisera.

		Agglutinin titers to each PSgc type strain							
		
Antiserum	Absorbing	G2351	G5305	G5306	G5307	G5308	G5310	G5313	G5319
	antigen(s)	(PSgc1)	(PSgc2)	(PSgc3)	(PSgc4)	(PSgc5)	(PSgc6)	(PSgc7)	(PSgc8)
PSgc1	G5306, G5313	640	–	–	–	–	–	–	–
PSgc2	G5307	–	160	–	–	–	–	–	–
PSgc3	G5313	–	–	320	–	–	–	–	–
PSgc4	G5305, G5313, G5319	–	–	–	320	–	–	–	–
PSgc5	G5306	–	–	–	–	160	–	–	–
PSgc6	–	–	–	–	–	–	320	–	–
PSgc7	G2351, G5306, G5319	–	–	–	–	–	–	320	–
PSgc8	G5306, G5307	–	–	–	–	–	–	–	320


### Development of a Multiplexed Luminex-Based Array

Compared with nucleotide sugar precursor synthesis genes, glycosyltransferase genes and processing genes are very heterogeneous among serotypes owing to the wide range of possible sugar linkages, and they thereby provide the potential as genetic targets for serotypes (Li and Reeves, 2000). Furthermore, in contrast to the glycosyltransferase gene, the *wzy* gene encoding polymerase and the *wzx* gene encoding flippase are much more highly serotype determinative (Ballmer et al., 2007). Consequently, all primers and probes, except those for PSgc4, were designed based on *wzy*. For PSgc4, as it processes no *wzy* or *wzx* gene and as all three glycosyltransferase genes give no identity to any sequences via BLAST search, we selected *GT2* as the specific gene (**Table 3**).

**Table 3:** Primers and probes used in the multiplex Luminex-based assay.

PSgc type	Primer sequence (5′–3′)		Size (bp)	Probe sequence
			
	Forward	Reverse		
PSgc1	TTGATTACTGGCGTTGGC	TTCACCCACAGACTGTTCC	192	GATGGCTTCAAATCAAGTAGT
PSgc2	TTGTTCTGGAATCTGGGTT	CGCATCTTGAAGGGTTGTA	272	AGCACTATACATACCGATTAAA
PSgc3	TTTGGTCGTACTGGTTAGG	AATCCCACCATTCAAACG	286	GCTATTACCAAATGCGAGTAG
PSgc4	ATAGAGACGGTAAAGAAAGTAA	TCCCGATTTCTGTCCTTC	325	ACTGGGATACATGGTTGCG
PSgc5	ATACTGGTGAGATGGAGGAA	CCCGCCATTTAGTCTTCC	276	ATACGCAGCATTTAAGAGAGA
PSgc6	TTTCATCGCCCATTACCT	TTGAACAGCCAAGGAAAT	246	AAGGTTCAGTTGGGTTACGC
PSgc7	TTATTTGTTGGACTGGCTAT	CAGGCGAATCAATAAAGG	232	ACCAGGTTTGGCAAGACA
PSgc8	TTCAGGCTTGGGAATAGA	TGAGGCTTGCTATTCTACG	305	TTTCGTGGGACAAACGTAGA


**FIGURE 2:**
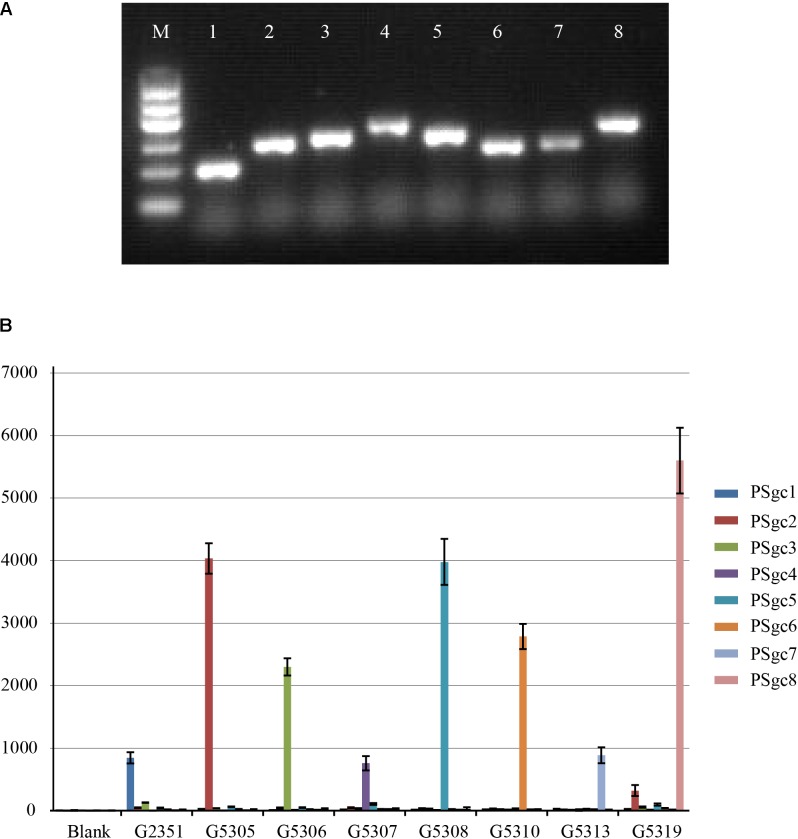
Results of multiplex Luminex-based array. **(A)** Multiplex PCR of type strains representing each PSgc type. M, DNA Marker I (600, 500, 400, 300, 200, and 100 bp); 1, G2351; 2, G5305; 3, G5306; 4, G5307; 5, G5308; 6, G5310; 7, G5313; 8, G5319. **(B)** Specific detection based on the Luminex-based array. Biotin-labeled PCR products were separated by probe-coupled beads, and the hybridization signals are presented in terms of median fluorescence intensity (MFI) on the *y*-axis. Each sample representing the corresponding type strain is indicated on the *x*-axis. Data are combined from three independent experiments with error bars representing the standard deviation.

Twenty-three *E. aerogenes* strains carrying PSgc1-8 and other pathogenic bacteria, including *Escherichia coli* (*n* = 2), *Salmonella* spp. (*n* = 2), *Shigella* spp. (*n* = 2), *Klebsiella pneumoniae* (*n* = 2), *Klebsiella oxytoca* (*n* = 2), *Enterobacter cloacae* (*n* = 1), *Enterobacter sakazakii* (*n* = 1), *Vibrio cholerae* (*n* = 2), *Vibrio parahaemolyticus* (*n* = 1), and *Citrobacter freundii* (*n* = 1), were used to assess the specificity of our multiplexed Luminex-based array. Multiplex PCR produced only a single band of the expected size for each type strain, and no non-specific amplicons were detected (**Figure 2A**). The subsequent Luminex-based array analyses indicated that each PSgc-specific probe detected the homologous strains correctly, and signals corresponding to heterologies or other pathogens were not observed (**Figure 2B**). A double-blind test with 97 clinical isolates that were obtained from the SCDC (i.e., hereafter referred to as Shanghai isolates) was performed. The results from these tests indicated that each isolate belonged to one of the eight PSgc types (PSgc1-8). Among these isolates, 32 (33%) contained PSgc4, 16 (16%) contained PSgc7, 12 (12%) contained PSgc 6, and 11 (11%) contained either PSgc2 or PSgc8, with other PSgc types each corresponding to <10% of the isolates. This result was confirmed to be correct by ABI sequencing of each single PCR amplicon. The limit of detection for each of the target strains was determined by examining six serial 10-fold dilutions (10 ng to 0.1 pg) of each type strain’s genomic DNA. As a result, the sensitivity of our assay using genomic DNA was 0.1 ng for each strain.

### Molecular Serotyping Allocation of *E. aerogenes* Strains Based on Publicly Available Genome Sequences

**FIGURE 3:**
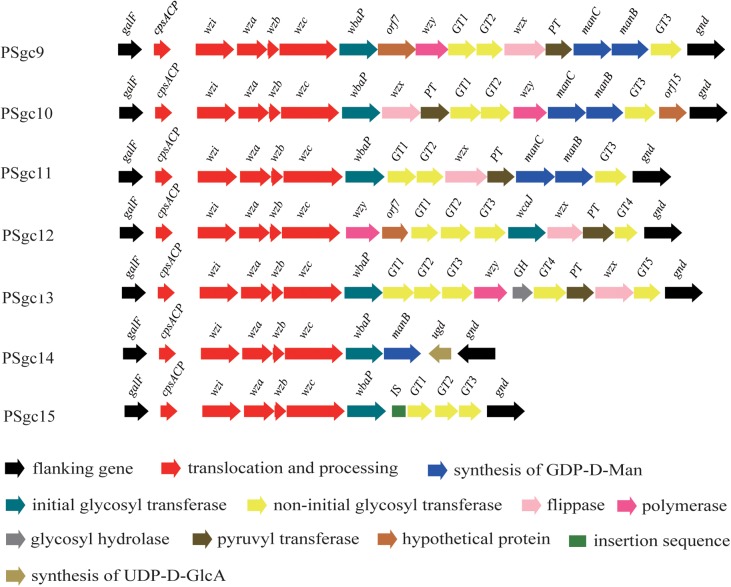
Schematic diagram of the seven novel putative PSgc types that were identified in 54 strains whose genomes were publicly available. Genes are represented by arrows and colored according to the gene key at the bottom with gene names indicated above each arrow.

Putative PSgc regions were extracted and identified from 86 *E. aerogenes* genomes that were obtained from the GenBank database. Thirty-two strains contained one of the seven PSgc types described above (PSgc3 was only found in G5306). In addition, another seven PSgc types (PSgc9-15) were distributed among the remaining 54 isolates (**Figure 3** and Supplementary Table 2). Details for all ORFs corresponding to these seven types are provided in Supplementary Table 3. Thus, our combined analyses identified a total of 15 PSgc types within the *E. aerogenes* molecular serotyping scheme. Among the 86 isolates, 37 (43%) contained PSgc10, followed by PSgc4 (12 of 86) and PSgc7 (7 of 86). Since the majority (74%) of the 86 strains were isolated from the United States and the remainder originated from other or unknown regions, the strains are tentatively referred to as “United States isolates” in the following discussion.

Within the 15 PSgcs, several anomalies were observed, including the following: (i) neither *wzx* nor *wzy* was annotated in PSgc4, PSgc14, or PSgc15, there was no *wzx* gene in PSgc7, and there was no *wzy* gene in PSgc2 or PSgc11. This atypical feature has been reported in *Klebsiella* capsular K11 and K34 without the *wzx* gene and in K29 and K50 without the *wzy* gene (Pan et al., 2015). In *Salmonella* serogroups A, B, and D1, the *wzy* genes also map far from the O-antigen gene cluster (Wang et al., 2002). Thus, it is likely that in the isolates with the above PSgc types, the functional *wzx/wzy* gene(s) are located outside of the PSgc region. However, they are difficult to annotate because of their low similarities to their counterparts, and further studies should be performed to confirm our hypothesis. (ii) In PSgc14, only *manB* was annotated. To our knowledge, this is the first evidence that *manC* and *manB* do not coexist in a gene cluster. (iii) It is noteworthy that the *ugd* and *gnd* genes are transcribed in the opposite direction in PSgc14 and that *ugd* is located at the 3′ end, meaning that there is probably a promoter at the end of the gene cluster.

## Discussion

In the present study, we developed a molecular serotyping system for *Enterobacter aerogenes* that is based on the PSgc region, which correlated perfectly with the conventional antigenic scheme. Hence, from the serotyping perspective, molecular PSgc-based typing and immunological serology are equally valid.

Differences in serotype distribution typically occur between geographical regions. For instance, among the genus *Shigella*, *S. flexneri* is globally and traditionally isolated frequently in resource-poor countries, with predominant serotypes including *S. flexneri* 2a, 3a, and 6, whereas *S. sonnei* is detected most commonly in high-income regions (Thompson et al., 2015). Using the molecular serotyping scheme presented here, we revealed the occurrence of diverse clusters of *E. aerogenes* serotypes between far-distant regions (Supplementary Figure 3). Strains containing the PSgc4 and PSgc7 types were the dominant serotypes among Shanghai isolates, while those with PSgc10 comprised the majority of United States isolates. It has been proposed that as one of the important components exposed on the cell surface, the bacterial surface polysaccharide is highly immunogenic and therefore subject to intense selection by the host immune system (Reeves and Wang, 2002). This selection probably accounts for the maintenance of surface polysaccharide diversity and offers certain serotype(s) selective advantages in specific niches of different hosts. Overall, our findings suggest an important distinction for clinical microbiologists, infectious disease specialists, and infection control agencies, although more data should be gathered to confirm these results.

In this study, we prepared antisera for each PSgc type strain and performed agglutination tests, providing evidence of the reliability of our molecular serotyping scheme. However, several disadvantages exist in the development and application of serotyping antisera and should be noted. First, the development of an antigenic scheme is sophisticated and time consuming (Orskov et al., 1977; Pan et al., 2015). Second, serotyping is labor-intensive, and only a few reference laboratories have the capacity to perform the analyses necessary to identify serotypes (Ballmer et al., 2007; Lacher et al., 2014). In addition, conventional serological assays that are based on agglutination reactions are also limited by the risks of the high prevalence of non-typeable isolates due to the expression of novel polysaccharide forms, which is common in clinical isolates (Jenney et al., 2006; Thrane et al., 2016; Guo et al., 2017). The development of NGS technologies and automated data analysis pipelines has led to a number of proof-of-concept studies. These studies have suggested the possibility of increased resolution of the existing typing methods and potential advantages in public health microbiology for outbreak investigation and surveillance (Dallman et al., 2015; Joensen et al., 2015; Zhang et al., 2015; Thrane et al., 2016). Moreover, a few practical genomics-based approaches have been reported for the typing of bacteria and pathogen surveillance (den Bakker et al., 2014; Dallman et al., 2015; Kwong et al., 2016). However, a few shortcomings of NGS seem insurmountable for the routine detection and identification of pathogens over a short period of time including (i) the need for pure isolate cultures to obtain enough quality genomic DNA (usually 50 ng to 1 μg depending on different sequencing platforms); (ii) the lengthy turnaround time for library preparation and NGS platform sequencing runs; and (iii) the relatively high cost per isolate genome sequence. Consequently, typing methods that are based on PSgc-specific gene(s) are likely to be widely applied and economically viable alternatives for the foreseeable future. These methodologies, including RFLP, multiplex PCR, high throughput real-time PCR, and microarrays, have been widely utilized to identify serotypes based on sero-specific genes in major pathogens, including *E. coli* (Coimbra et al., 2000; Botkin et al., 2012), *Streptococcus pneumonia*e (Azzari et al., 2010), *Salmonella* spp. (Guo et al., 2013) and *Streptococcus suis* (Bai et al., 2015). The Luminex-based array system is a multiplexed microsphere-based suspension system that offers a molecular diagnostic platform and provides an open and attractive approach for simultaneous, high-throughput and multiplex detection of up to 100 targets in protein and nucleic acid studies (Yan et al., 2017). It has been approved by the US FDA for clinical diagnosis and has been used in various applications (Dunbar, 2006; Liu et al., 2011; Glushakova et al., 2015; Silbereisen et al., 2015). Although the assay presented here only targeted *E. aerogenes* strains of types PSgc1-8, which appear to be more common in Shanghai and its surrounding environs, thus highlighting a limitation of the analysis (i.e., strain availability), the detection range of our assay could easily be expanded, as PSgc data can be obtained without limitation. Indeed, evaluation and revisiting of the serotyping system presented here should be performed as more isolates become available in the future. Regardless, our method shows significant potential for the characterization and epidemiological surveillance of this emerging pathogen.

We propose that all of the putative PSgc types identified in this study are responsible for the biosynthesis of capsular (K) antigens of *E. aerogenes*. The reasons for this supposition are as follows: (i) a conserved gene set, *cpsACP-wzi-wza-wzb-wzc*, is located at the 5′ end of each PSgc, which shares the typical features of most K antigen gene clusters in the genus *Klebsiella*, which is closely related to *E. aerogenes*, and the group 1 capsule gene clusters of *E. coli* (Whitfield, 2006; Pan et al., 2015). (ii) In the genome of *E. aerogenes* CAV1320, which contained PSgc5, another genetic region (loci: ABY61_23370 to ABY61_23395) containing six genes (*wzm*, *wzt*, *glf*, and *GT*1-3) was found upstream of *hisI*, which resembled an O antigen gene cluster (Supplementary Figure 4). This region has been purported to have been introduced into an *E. coli* strain by an IS element, forming the O62 serotype in our recent study (Hou et al., 2017). We also examined the putative O antigen gene cluster among the strains for which whole genomes were available. Ten types of putative O-antigen gene clusters were characterized and distributed among 105 genomes, except for another four incomplete genomes due to poor sequencing (data not shown).

Seven of eight antisera gave slight serological cross-reactivity with heterologous antigens before absorption. It is hard to predict whether this is attributable to the similar polysaccharides, as no chemical structure has ever been reported. However, the PSgc types of each strain are quite different, meaning probably that no similar structures are shared among them. We therefore propose that the weak cross-reaction is due to the presence of common epitopes of other cell surface components. More detailed studies of each PSgc and the corresponding polysaccharide structures should be performed, and the correlation between them will be necessary to completely elucidate the heredity and evolution of *E. aerogenes*. In this regard, the results presented here provide a valuable framework from which to further assess the evolution of *E. aerogenes* surface polysaccharide structures in response to host and bacteriophage interactions.

## Author Contributions

XG, BL, and LF conceived the project. XG and MW prepared the strain samples, preformed the sequence analyses, and developed the molecular serotyping system. LW conducted the bioinformatics analyses. YW and TC developed the conventional antigenic scheme. PW developed the multiplexed Luminex-based array. MC performed the double-blind test. XG, BL, and LF prepared the manuscript. All authors read and approved the final manuscript.

## Conflict of Interest Statement

The authors declare that the research was conducted in the absence of any commercial or financial relationships that could be construed as a potential conflict of interest.

## Footnotes

1 www.ncbi.nlm.nih.gov/genbank

2 pfam.sanger.ac.uk

3 http://www.cbs.dtu.dk/services/TMHMM-2.0/
