# Supplementary material for: Establishment of a Molecular Serotyping Scheme and a Multiplexed Luminex-Based Array for Enterobacter aerogenes
Source: Front Microbiol. 2018 Mar 19;9:501. doi: 10.3389/fmicb.2018.00501 (PMC5867348; doi:10.3389/fmicb.2018.00501)
Supplement: Supplementary file 1 [file Table_1.PDF]

Table S1. The *Enterobacter aerogenes* strains used for sequence analysis in this study

Strain	Lab collection No.	source[a]	sample	Whole genome accession No.
	G2351	SCDC		
	G5305	SCDC		
	G5306	SCDC		
	G5307	SCDC		
	G5308	SCDC		
	G5309	SCDC		
	G5310	SCDC		
	G5311	SCDC		
	G5312	SCDC		
	G5313	SCDC		
	G5314	SCDC		
	G5316	SCDC		
	G5319	SCDC		
	G5832	SCDC		
	G5835	SCDC		
	G5836	SCDC		
	G5837	SCDC		
	G5840	SCDC		
	G5844	SCDC		
12058	G5982	DSMZ		
30053	G5983	DSMZ		
29940	G5987	ATCC		
13048	G5989	ATCC		
KCTC 2190				NC_015663
EA1509E		Marseille, France	Blood	NC_020181
CAV1320		Virginia, USA	Perirectal	NZ_CP011574
FGI35			Human Airways	NZ_KB911089
UCI 48		Irvine, CA, USA	Sputum	NZ_KI973098
UCI 47		Irvine, CA, USA	Sputum	NZ_KI973103
UCI 46		Irvine, CA, USA	Cerebro-spinal fluid	NZ_KI973107
UCI 45		Irvine, CA, USA	Sputum	NZ_KI973115

UCI 28	Irvine, CA, USA	Drainage	NZ_KI973148
UCI 27	Irvine, CA, USA	Blood	NZ_KI973151
UCI 16	Irvine, CA, USA	Blood	NZ_KI973159
UCI 15	Irvine, CA, USA	Aspirate	NZ_KI973163
MGH 61	Boston, USA	Blood	NZ_KK736187
MGH 62	Boston, USA	Wound	NZ_KK736200
MGH 77	Boston, USA	Wound	NZ_KK736204
MGH 78	Boston, USA	Urine	NZ_KK736212
CDC UA0804-01			NZ_KN150798
UCI 89			NZ_KQ087571
UCI 90			NZ_KQ087584
UCI 97			NZ_KQ087587
ND17	USA	Excreted bodily substance	NZ_JUHZ01000201
33850			NZ_LAAO01000014
35007	New York, USA		NZ_JZZY01000014
42193	New York, USA		NZ_JZYI01000020
28944	New Jersey, USA		NZ_LAAX01000066
32540	New Jersey, USA		NZ_LAAU01000013
35003	New York, USA		NZ_LAAC01000067
35005	New York, USA		NZ_LAAA01000094
35006	New York, USA		NZ_JZZZ01000026
35715	Florida, USA		NZ_JZYV01000011
44247	New York, USA		NZ_JZXY01000010
B3	Malaysia	Bitter gout	NZ_JSWV01000001

GN03019	USA	Bodily fluid	NZ_LDBQ01000054
GN02173	USA	Bodily fluid	NZ_LDBB01000048
GN02286	USA	Bodily fluid	NZ_LDBD01000064
GN02326	USA	Bodily fluid	NZ_LDBE01000044
GN02278	USA	Bodily fluid	NZ_LDBC01000052
GN02355	USA	Bodily fluid	NZ_LDBG01000049
GN02384	USA	Bodily fluid	NZ_LDBH01000048
GN02420	USA	Bodily fluid	NZ_LDBI01000050
GN04690	USA	Bodily fluid	NZ_LDBW01000064
GN03543	USA	Bodily fluid	NZ_LDBR01000049
GN02079	USA	Bodily fluid	NZ_LDAY01000055
GN02126	USA	Bodily fluid	NZ_LDAZ01000048
GN02464	USA	Bodily fluid	NZ_LDBJ01000050
GN02329	USA	Bodily fluid	NZ_LDBF01000053
GN02499	USA	Bodily fluid	NZ_LDBK01000068
GN02509	USA	Bodily fluid	NZ_LDBL01000053
GN02694	USA	Bodily fluid	NZ_LDBN01000048
GN02525	USA	Bodily fluid	NZ_LDBM01000098
GN03688	USA	Bodily fluid	NZ_LDBS01000059

GN03927	USA	Bodily fluid	NZ_LDBU01000044
GN03959	USA	Bodily fluid	NZ_LDBV01000052
GN02761	USA	Bodily fluid	NZ_LDBO01000067
GN03732	USA	Bodily fluid	NZ_LDBT01000048
GN04835	USA	Bodily fluid	NZ_LDBY01000058
GN05253	USA	Bodily fluid	NZ_LDCA01000058
GN05224	USA	Bodily fluid	NZ_LDBZ01000056
GN04794	USA	Bodily fluid	NZ_LDBX01000063
UCI 98			NZ_LESY01000001
1281_EAER	Washington¸ USA		NZ_JVVQ01000100
170_EAER	Washington¸ USA		NZ_JVRL01000021
965_EAER	Washington¸ USA		NZ_JULT01000023
SMART_350	Canada		NZ_LPQH01000023
SMART_429	Tunisia		NZ_LPQB01000034
SMART_543	USA		NZ_LPPU01000034
SMART_773	Brazil		NZ_LPPE01000045
SMART_774	Brazil		NZ_LPPD01000034
SMART_888	Guatemala		NZ_LPOU01000034
SMART_1060	Saudi Arabia		NZ_LPOM01000034

GN02710	USA		NZ_LRCB01000001
GN05809	USA		NZ_LRDA01000001
GN05782	USA		NZ_LRCY01000001
GN05748	USA		NZ_LRCW01000001.1
SMART_1372	Colombia	Urine	NZ_LRJR01000001
SMART_1249	Turkey	Abdomen	NZ_LRIT01000001
SMART_1248	Turkey	Urine	NZ_LRIU01000001
1277_EAER	Washington¸ USA		NZ_JVVV01000065
1282_EAER	Washington¸ USA		NZ_JVVP01000067
1019_EAER	Washington¸ USA		NZ_JWGB01000134
1278_EAER	Washington¸ USA		NZ_JVVU01000074
151_EAER	Washington¸ USA		NZ_JVSF01000030
86_EAER	USA		NZ_JUQG01000166
225_EAER	USA		NZ_JVPH01000874
GN02770	USA	Bodily fluid	NZ_LDBP01000079
G7	France		NZ_CP011539

a SCDC, Shanghai municipal center for disease control and prevention; ATCC, American type culture collection; DSMZ, German collection of microorganisms and clutures.
