# Supplementary material for: Establishment of a Molecular Serotyping Scheme and a Multiplexed Luminex-Based Array for Enterobacter aerogenes
Source: Front Microbiol. 2018 Mar 19;9:501. doi: 10.3389/fmicb.2018.00501 (PMC5867348; doi:10.3389/fmicb.2018.00501)
Supplement: Supplementary file 2 [file Table_2.PDF]

Table S2. PSgc allocation and the corresponding accession numbers

PSgc form	Type strain	Other strains	Accession numbers
PSgc1	G2351	GN02384; GN02499; 151_EAER;SMART_543	MF687352
PSgc2	G5305	28944; GN04690; GN05224;SMART_429	MF687353
PSgc3	G5306	**N/A**	MF687354
PSgc4	G5307	GN02278; GN02286; GN02525; GN03543; GN04794; MGH_61;UCI_27; UCI_28;SMART_1372;SMART_774;SMSRT_773;SMART_350; G5311;G5312;G5316;G5832;G5837;G5844; G5987	MF687355
PSgc5	G5308	CAV1320; G5309;G5835	MF687356
PSgc6	G5310	G5314;G5836;G5840; 35003; ND17	MF687357
PSgc7	G5313	G5983; G5989; GN02710; KCTC2190; FGI35;GN02420;GN03732; GN03688	MF687358
PSgc8	G5319	G5982; SMART_1060; UCI_89;CDC UA0804-01	MF687359
PSgc9	86_EAER	965_EAER;GN02770;GN04835;GN05253	MF687360
PSgc10	170_EAER	1019_EAER;1277_EAER;1278_EAER;1281_EAER;1282_EAER;32540;35715;44247;GN02126;GN02173;GN02326;GN02329;GN02355;GN02464;GN03019;GN03927;MGH_62;MGH_77;MGH_78;UCI_15;UCI_16;UCI_46;UCI_47;UCI_48;UCI_90;UCI_97;UCI_98;SMART1249;G7;SMART1248;SMART888;GN05809;GN03959;GN05782;GN05748;GN02079	MF687361
PSgc11	225_EAER	GN02761; 35005;35006;35007;GN02509; GN02694	MF687362
PSgc12	33850	**N/A**	MF687363
PSgc13	42193	B3	MF687364
PSgc14	EA1509E	**N/A**	MF687365
PSgc15	UCI_45	**N/A**	MF687366
