# Supplementary material for: Establishment of a Molecular Serotyping Scheme and a Multiplexed Luminex-Based Array for Enterobacter aerogenes
Source: Front Microbiol. 2018 Mar 19;9:501. doi: 10.3389/fmicb.2018.00501 (PMC5867348; doi:10.3389/fmicb.2018.00501)
Supplement: Supplementary file 3 [file Table_3.PDF]

Table S3. Characteristics of the ORFs in all putative PSgcs

E. aerogenes PSgc1							
Orf no.	Gene name	Position of gene	G+C content (%)	Conserved domain(s)	Similar protein(s), strain(s) (Genbank accession No.)	%Identical /%Similar (total No. of aa)	Putative function of protein
1	*cpsACP*	1..630	50.79	PAP2 superfamily (PF01569.18) *E* value=4.6e-09	acid phosphatase, [*Klebsiella oxytoca*] (SAQ01243)	73/84(209)	Acid phosphatase
2	*wzi*	1565..2998	49.16	Caps_assemb_Wzi (PF14052.3) *E* value= 1.6e-126	surface assembly of capsule [*Klebsiella pneumoniae*] (BAO24050)	88/93(477)	Capsule assembly protein Wzi
3	*wza*	3138..4277	42.11	Polysaccharide biosynthesis/export protein (PF02563.13) *E* value= 3.4e-28	polysaccharide export protein, [*Klebsiella pneumoniae*] (WP_032441376)	92/96(379)	Polysaccharide biosynthesis/export protein
4	*wzb*	4277..4711	40.00	LMWPc (PF01451.18) *E* value= 1e-39	protein tyrosine phosphatase [*Klebsiella oxytoca*] (AEX06753）	73/81(144)	Low molecular weight protein-tyrosine-phosphatase Wzb
5	*wzc*	4728..6896	36.84	Wzz (PF02706.12) *E* value=1.4e-46	Tyrosine-protein kinase Wzc,	99/99(722)	Putative tyrosine-protein kinase in cps region

					[*Enterobacter cloacae*] (SAJ11239.1)		
6	*wzy*	6971..8248	31.61		O-antigen ligase like membrane protein, [*Serratia marcescens*] (CUY98466)	46/62(425)	Polymerase
7	*GT1*	8273..9349	31.2	Glycos_transf_1 (PF00534.17) *E* value= 1.3e-16	group 1 glycosyl transferase [*Enterobacter cloacae*] (SAJ11230)	99/99(358)	Group 1 glycosyl transferase
8	*GT2*	9346..10554	33.91	Glyco_trans_1_4 (PF13692.3) *E* value=1.3e-14	glycosyl transferase, [*Serratia proteamaculans*] (ABV40692)	51/69(402)	Glycosyl transferase group 1
9	*PT*	10574..11650	29.71	PS_pyruv_trans (PF04230.10) *E* value=7.3e-21	polysaccharide pyruvyl transferase CsaB [*Enterobacter cloacae*] (SAJ11219)	97/99(358)	Polysaccharide pyruvyl transferase
10	*GT3*	11661..12833	32.48	Glycos_transf_1 (PF00534.17) *E* value= 1.7e-12	glycosyl transferase 1, [*Serratia*] (WP_048762193)	53/71(390)	Glycosyl transferases group 1
11	*wzx*	12843..14300	33.13	Polysacc_synt_3	Flippase,	59/79(485)	Flippase

				(PF13440.3)<br>*E* value= 2.8e-86	[*Klebsiella sp.]*<br>(BAT24267)		
12	*GT4*	14297..15358	30.32	Glyco_transf_4<br>(PF13439.3)<br>*E* value= 7.4e-10	glycosyl transferase,<br>[*Serratia proteamaculans*]<br>(WP_012006025)	60/78(353)	Glycosyl transferase
13	*HG*	15380..16333	33.33	Cellulase<br>(PF00150.15)<br>*E* value= 2.2e-8	glycosyl hydrolase,<br>[*Klebsiella sp*.]<br>(BAT24275)	44/63(317)	Glycosyl hydrolase
14	*wcaJ*	16493..17878	33.84	Bac_transf<br>(PF02397.13)<br>*E* value= 5.2e-57	UDP-glucose lipid carrier transferase<br>[*Klebsiella pneumonia*]<br>(KYL83484)	66/79(461)	UDP-glucose:undecaprenyl-phosphate glucose-1-phosphate transferase

E. aerogenes PSgc2							
Orf no.	Gene name	Position of gene	G+C content (%)	Conserved domain(s)	Similar protein(s), strain(s) (Genbank accession No.)	%Identical /%Similar (total No. of aa)	Putative function of protein
1	*cpsACP*	1..630	50	PAP2 superfamily (PF01569.18) *E* value=4.6e-09	acid phosphatase, [*Klebsiella oxytoca*] (SAQ01243)	73/84 (209)	Acid phosphatase
2	*wzi*	1563..3005	49.34	Caps_assemb_Wzi (PF14052.3) *E* value= 1.6e-126	surface assembly of capsule [*Klebsiella pneumoniae*] (BAO24050)	88/93 (480)	Capsule assembly protein Wzi
3	*wza*	3120..4259	41.49	Polysaccharide biosynthesis/export protein (PF02563.13) *E* value= 3.4e-28	polysaccharide export protein, [*Klebsiella pneumoniae*] (WP_032441376)	92/96(379)	Polysaccharide biosynthesis/export protein
4	*wzb*	4259..4693	42.3	LMWPc (PF01451.18) *E* value= 1e-39	protein tyrosine phosphatase [*Klebsiella oxytoca*] (AEX06753)	73/81(144)	Low molecular weight protein-tyrosine-phosphatase Wzb
5	*wzc*	4711..6867	37.18	Wzz (PF02706.12) *E* value=1.4e-46	Tyrosine-protein kinase Wzc, [*Enterobacter cloacae*] (SAJ11239.1)	99/99 (718)	Putative tyrosine-protein kinase in cps region
6	*wzy*	6937..8229	31.71		Lipid A core-O-antigen ligase and related enzymes, *[Serratia marcescens*] (CUY98466)	43/62(430)	Polymerase

7	*GT1*	8241..9356	31.09	Glycos_transf_1 family (PF00534.17) 8.7e-21	glycosyl transferase family protein, [*Klebsiella sp*. 6168] (BAT23615)	52/67(371)	Glycosyl transferase group 1
8	*wzx*	9424..10953	32.55		Flippase, [Klebsiella sp. 7444] (BAT23657)	63/81(509)	Flippase
9	*PT*	10970..11950	35.68	PS_pyruv_trans family (PF04230.10) 1.3e-14	Polysaccharide pyruvyl transferase, [*Serratia marcescens*] (CVE11324)	53/70(326)	Polysaccharide pyruvyl transferase
10	*manC*	12035..13456	38.12	NTP_transferase family (PF00483.20) 2.8e-72	Mannose-1-phosphate guanylyltransferase, [*Enterobacter aerogenes*] (KJF84403)	71/85(473)	Mannose-1-phosphate guanylyltransferase
11	*manB*	13472..14848	36.82	PGM_PMM_I domain (PF02878.13) 2.4e-32	Phosphomannomutase, [*Klebsiella pneumoniae*] (WP_032733344)	81/90(458)	Phosphomannomutase/phosphoglucomuta se
12	*GT2*	14906..16060	35.76	Glycos_transf_1 family (PF00534.17) 9e-27	glycosyl transferase family 1 [*Klebsiella pneumoniae*] (WP_025403910.1)	78/88(384)	alpha-(1-6)-phosphatidylinositol monomannoside mannosyltransferase
13	*wbaP*	16122..17549	35.57	Bac_transf family (PF02397.13) 5.3e-57	UDP-phosphate galactose phosphotransferase [*Klebsiella oxytoca*] (WP_049080021)	71/82(475)	Undecaprenyl-phosphate galactose phosphotransferase
14	*GT3*	17665..18714	33.05	Glycos_transf_1 family	glycosyltransferase, group 1	57/77(349)	N-acetylgalactosamine-N,N'-diacetylbacill

				(PF00534.17)<br>3.1e-27	family protein [*Klebsiella pneumoniae* MRSN 1319]<br>(KGT62405.1)		osaminyl-diphospho-undecaprenol 4-alpha-N-acetylgalactosaminyltransferas e
15	*HG*	18734..19765	36.92	Cellulase domain<br>(PF00150.15)<br>6.5e-6	glycoside hydrolase [*Klebsiella sp*. 1680/49]<br>(BAT23450)	46/64(343)	Glycoside hydrolase
16	*AT*	19886..21034	34.46	Acyl_transf_3 family<br>(PF01757.19)<br>3e-27	acyltransferase [*Raoultella ornithinolytica*]<br>(WP_041146355)	70/82(382)	O-acetyltransferase OatA

E. aerogenes PSgc3							
Orf no.	Gene name	Position of gene	G+C content (%)	Conserved domain(s)	Similar protein(s), strain(s) (Genbank accession No.)	%Identical /%Similar (total No. of aa)	Putative function of protein
1	*cpsACP*	1..630	50.79	PAP2 superfamily (PF01569.18) *E* value=4.6e-09	acid phosphatase, [*Klebsiella oxytoca*] (SAQ01243)	73/84 (209)	Acid phosphatase
2	*wzi*	1563..3002	47.99	Caps_assemb_Wzi (PF14052.3) *E* value= 1.6e-126	surface assembly of capsule [*Klebsiella pneumoniae*] (BAO24050)	88/93 (479)	Capsule assembly protein Wzi
3	*wza*	3116..4252	43.71	Polysaccharide biosynthesis/export protein (PF02563.13) *E* value= 3.4e-28	polysaccharide export protein, [*Klebsiella pneumoniae*] (WP_032441376)	92/96 (378)	Polysaccharide biosynthesis/export protein
4	*wzb*	4256..4690	46.21	LMWPc (PF01451.18) *E* value= 1e-39	protein tyrosine phosphatase [*Klebsiella oxytoca*] (AEX06753)	73/81 (144)	Low molecular weight protein-tyrosine-phosphatase Wzb
5	*wzc*	4709..6877	40.89	Wzz (PF02706.12) *E* value=1.4e-46	Tyrosine-protein kinase Wzc, [*Enterobacter cloacae*] (SAJ11239.1)	99/99(722)	Putative tyrosine-protein kinase in cps region
6	*wbaP*	6941..8389	38.87	Bac_transf family (PF02397.13) *E* value=7.8e-56	undecaprenyl-phosphate galactose phosphotransferase WbaP [*Escherichia coli*] (ref|WP_000149073.1|)	82/90(482)	Undecaprenyl-phosphate galactose phosphotransferase
7	*GT1*	8408..9499	32.33	Glyco_trans_1_4 domain (PF13692.3)	glycosyltransferase [*Klebsiella*	44/63(363)	Glycosyltransferase

				E value=1.4e-6	*pneumoniae*] (BAO24069)		
8	*wzy*	9519..10790	32.86	EpsG family (PF14897.3) *E* value=4.7e-16	Wzy [*Shigella boydii*] (gb|ABI55342.1|)	26/44(423)	Polymerase
9	*orf9*	10780..11586	35.07	Glyco_hydro_cc family (PF11790.5) *E* value=5.1e-48	hypothetical protein [*Klebsiella sp.* 1702] BAT23785	63/79(268)	Hypothetical protein
10	*GT2*	11625..12857	32.44	Glyco_trans_1_4 domain (PF13692.3) *E* value=3.1e-13	glycosyl transferase [*Klebsiella sp.* 1702] (BAT23786)	70/85 (410)	Glycosyl transferase
11	*GT3*	12850..13941	34.34	Glycos_transf_1 family (PF00534.17) *E* value=3.5e-17	glycosyl transferases group 1 family protein [*Burkholderia cenocepacia*] (AIO39610)	32/50(363)	N-acetylgalactosamine-N, N'-diacetylbacillosaminyl-diphospho-undecaprenol 4-alpha-N-acetylgalactosaminyltransferase
12	*manC*	14250..15665	43.64	NTP_transferase family (PF00483.20) *E* value=1.6e-73	mannose-1-phosphate guanylyltransferase/mannose-6-phosphate isomerase [*Klebsiella pneumoniae*] (WP_032412638)	90/96(471)	Mannose-1-phosphate guanylyltransferase 1
13	*manB*	15687..17057	44.42	PGM_PMM_I domain (PF02878.13) *E* value=2.6e-33	phosphomannomutase [*Klebsiella pneumoniae*] (WP_021313304)	87/94(456)	Phosphomannomutase/phosphoglucomutase
14	*vioA*	17157..18266	37.03	DegT_DnrJ_EryC1 domain (PF01041.14) *E* value=3e-103	dTDP-4-amino-4,6-dideoxy-D-glucose transaminase [*Thiomonas sp.* CB3]	68/81(369)	dTDP-4-amino-4,6-dideoxy-D-glucose transaminase

					(CQR41857)		
15	*vioB*	18254..18832	36.96	Hexapep domain (PF00132.21) *E* value=8e-6	dTDP-4-amino-4,6-dideoxy-D-glucose acyltransferase [*Thiomonas sp.* CB3] (CQR41856)	59/80(192)	dTDP-4-amino-4,6-dideoxy-D-glucose acyltransferase
16	*GT4*	18832..19980	35.16	Glyco_transf_56 family (PF07429.8) *E* value=4.2e-13	4-alpha-L-fucosyltransferase [*Serratia marcescens*] (WP_015673931)	49/67(382)	TDP-N-acetylfucosamine:lipid II N-acetylfucosaminyltransferase
17	*wzx*	19991..21394	34.76	Polysacc_synt family (PF01943.14) *E* value=2.7e-17	polysaccharide biosynthesis protein [*Citrobacter freundii*] (WP_060854689)	41/61(467)	Flippase
18	*rmlB*	21533..22597	42.44	GDP_Man_Dehyd domain (PF16363.2) *E* value=6.5e-94	dTDP-glucose 4,6-dehydratase [*Enterobacter aerogenes*] (WP_045389218)	88/94(354)	TDP-glucose 4,6-dehydratase 2
19	*rmlA*	22610..23479	39.89	NTP_transferase family (PF00483.20) *E* value=9.9e-68	glucose-1-phosphate thymidylyltransferase [*Enterobacter aerogenes*] (WP_032711985)	90/96(289)	Glucose-1-phosphate thymidylyltransferase 2

E. aerogenes PSgc4							
Orf no.	Gene name	Position of gene	G+C content (%)	Conserved domain(s)	Similar protein(s), strain(s) (Genbank accession No.)	%Identical /%Similar (total No. of aa)	Putative function of protein
1	*cpsACP*	1..630	50.63	PAP2 superfamily (PF01569.18) *E* value=4.6e-09	acid phosphatase, [*Klebsiella oxytoca*] (SAQ01243)	73/84(209)	Acid phosphatase
2	*wzi*	1564..2997	49.23	Caps_assemb_Wzi family (PF14052.3) *E* value= 1.6e-126	surface assembly of capsule [*Klebsiella pneumoniae*] (BAO24050)	88/93 (477)	Capsule assembly protein Wzi
3	*wza*	3117..4253	43.89	Polysaccharide biosynthesis/export protein family (PF02563.13) *E* value= 3.4e-28	polysaccharide export protein, [*Klebsiella pneumoniae*] (WP_032441376)	92/96 (378)	Polysaccharide biosynthesis/export protein
4	*wzb*	4244..4690	42.28	LMWPc domain (PF01451.18) *E* value= 1.4e-40	protein tyrosine phosphatase [*Klebsiella oxytoca*] (AEX06753)	73/81 (148)	Low molecular weight protein-tyrosine-phosphatase Wzb
5	*wzc*	4708..6870	37.59	Wzz family (PF02706.12) *E* value=1.4e-46	Tyrosine-protein kinase Wzc, [*Enterobacter cloacae*] (SAJ11239.1)	99/99 (720)	Putative tyrosine-protein kinase in cps region
6	*wbaP*	6967..8394	38.31	Bac_transf family (PFPF02397.13) *E* value=9.3e-58	undecaprenyl-phosphate galactose phosphotransferase WbaP [*Enterobacter aerogenes*] (WP_032708709)	99/100(475)	Undecaprenyl-phosphate galactose phosphotransferase

7	*GT1*	8437..9348	37.5	Glycos_transf_2 family (PF00535.23) 3.7e-14	rhamnosyltransferase [*Klebsiella oxytoca*] (WP_049080022)	72/86(303)	Glycosyl transferase family 2
8	*GT2*	9464..10330	36.1	Glycos_transf_2 family (PF00535.23) 9.8e-28	glycosyl transferase family 2 [*Enterobacter aerogenes*] (WP_015706052)	63/79(288)	GalNAc(5)-diNAcBac-PP-undecaprenol beta-1,3-glucosyltransferase
9	*GT3*	10355..11194	35.71	Glycos_transf_2 family (PF00535.23) 8.2e-7	glycosyl transferase family 2 [*Serratia rubidaea*] (WP_054304891)	(279)	Glycosyl transferase family 2

E. aerogenes PSgc5							
Orf no.	Gene name	Position of gene	G+C content (%)	Conserved domain(s)	Similar protein(s), strain(s) (Genbank accession No.)	%Identical /%Similar (total No. of aa)	Putative function of protein
1	*cpsACP*	1..630	51.11	PAP2 superfamily (PF01569.18) *E* value=4.6e-09	acid phosphatase, [*Klebsiella oxytoca*] (SAQ01243)	73/84(209)	Acid phosphatase
2	*wzi*	1563..3002	48.4	Caps_assemb_Wzi family (PF14052.3) *E* value= 1.6e-126	surface assembly of capsule [*Klebsiella pneumoniae*] (BAO24050)	88/93 (479)	Capsule assembly protein Wzi
3	*wza*	3113..4249	42.74	Polysaccharide biosynthesis/export protein family (PF02563.13) *E* value= 3.4e-28	polysaccharide export protein, [*Klebsiella pneumoniae*] (WP_032441376)	92/96 (378)	Polysaccharide biosynthesis/export protein
4	*wzb*	4252..4686	46.67	LMWPc domain (PF01451.18) *E* value= 1.4e-40	protein tyrosine phosphatase [*Klebsiella oxytoca*] (AEX06753)	73/81 (144)	Low molecular weight protein-tyrosine-phosphatase Wzb
5	*wzc*	4704..6872	40.43	Wzz family (PF02706.12) *E* value=1.4e-46	Tyrosine-protein kinase Wzc, [*Enterobacter cloacae*] (SAJ11239.1)	99/99 (722)	Putative tyrosine-protein kinase in cps region
6	*wbaP*	6966..8393	39.08	Bac_transf family (PFPF02397.13)	undecaprenyl-phosphate galactose phosphotransferase WbaP [*Klebsiella*	98/99(475)	Undecaprenyl-phosphate galactose phosphotransferase

				E value=9.3e-58	*pneumoniae*] (WP_050849632)		
7	*GT1*	8415..9509	31.32		glycosyltransferase [*Klebsiella pneumoniae*] (BAO24069)	42/62(364)	Glycosyl transferase
8	*wzy*	9517..10794	31.61	EpsG family (PF14897.3) *E* value=5.3e-18	Wzy [*Acinetobacter baumannii*] (AHB32687)	27/43(425)	Polymerase
9	*orf9*	10778..11596	33.09	Glyco_hydro_cc family (PF11790.5) *E* value=1.4e-46	hypothetical protein [*Klebsiella pneumoniae*] (WP_050849629)	99/99(272)	Function unkonwon
10	*GT2*	11620..12870	30.7	Glyco_trans_1_4 domain (PF13692.3) *E* value=3e-14	glycosyl transferase group 1 [*Serratia proteamaculans* 568] (gb|ABV40692.1|)	40/57(416)	Glycosyl transferase
11	*GT3*	12863..13957	32.24	Glycos_transf_1 family (PF00534.17) *E* value=7.1e-20	glycosyl transferase group 1 [*Burkholderia sp*. H160] (EEA01522)	31/51(364)	D-inositol-3-phosphate glycosyltransferase
12	*manC*	13961..15385	37.4	NTP_transferase family (PF00483.20) *E* value=4.7e-73	mannose-1-phosphate guanylyltransferase/mannose-6-phosphate isomerase [*Enterobacter aerogenes*] (WP_047740523)	100/100(474)	Mannose-1-phosphate guanylyltransferase 1
13	*manB*	15403..16779	37.4	PGM_PMM_I domain	phosphomannomutase [*Shimwellia blattae*]	78/87(458)	Phosphomannomutase/phosphoglucomutase

				(PF02878.13) *E* value=3.8e-33	(WP_002441829)		
14	*GT4*	16867..17793	34.41	Glycos_transf_2 family (PF00535.23) *E* value=5.8e-21	glycosyl transferase [*Klebsiella sp*. 1702] (BAT23790)	97/99(308)	Galactofuranosyl transferase GlfT1
15	*PT*	17867..18910	31.32	PS_pyruv_trans family (PF04230.10) *E* value=2.7e-21	exopolysaccharide pyruvyl transferase [*Bacillus subtilis*] (WP_003236435)	34/54(347)	Pyruvyl transferase
16	*wzx*	18975..20396		Polysacc_synt family (PF01943.14) *E* value=5.8e-18	flippase [*Klebsiella sp*. 313] (BAT23305)	65/81(473)	Flippase
17	*glf*	20433..21527	34.89	GLF family (PF03275.10) *E* value=2.6e-83	UDP-galactopyranose mutase [*Enterobacter aerogenes*] (WP_047740530)	100/100(364)	UDP-galactopyranose mutase

E. aerogenes PSgc6							
Orf no.	Gene name	Position of gene	G+C content (%)	Conserved domain(s)	Similar protein(s), strain(s) (Genbank accession No.)	%Identical /%Similar (total No. of aa)	Putative function of protein
1	*cpsACP*	1..630	50.32	PAP2 superfamily (PF01569.18) *E* value=4.6e-09	acid phosphatase, [*Klebsiella oxytoca*] (SAQ01243)	73/84(209)	Acid phosphatase
2	*wzi*	1551..2900	48.19	Caps_assemb_Wzi family (PF14052.3) *E* value= 1.6e-126	surface assembly of capsule [*Klebsiella pneumoniae*] (BAO24050)	88/93 (479)	Capsule assembly protein Wzi
3	*wza*	3103..4239	41.95	Polysaccharide biosynthesis/export protein family (PF02563.13) *E* value= 3.4e-28	polysaccharide export protein, [*Klebsiella pneumoniae*] (WP_032441376)	92/96 (378)	Polysaccharide biosynthesis/export protein
4	*wzb*	4230..4676	41.83	LMWPc domain (PF01451.18) *E* value= 1.4e-40	protein tyrosine phosphatase [*Klebsiella oxytoca*] (AEX06753)	73/81 (148)	Low molecular weight protein-tyrosine-phosphatase Wzb
5	*wzc*	4693..6855	38.51	Wzz family (PF02706.12) *E* value=1.4e-46	Tyrosine-protein kinase Wzc, [*Enterobacter cloacae*] (SAJ11239.1)	99/99 (720)	Putative tyrosine-protein kinase in cps region
6	*wbaP*	6923..8350	35.22	Bac_transf family (PFPF02397.13) *E* value=9.3e-58	undecaprenyl-phosphate galactose phosphotransferase WbaP [*Klebsiella pneumoniae*] (WP_050849632)	98/99(475)	Undecaprenyl-phosphate galactose phosphotransferase

7	*wzy*	8396..9658	34.28		Lipid A core-O-antigen ligase and related enzymes [*Serratia marcescens*] (CVG90993)	36/60(420)	Polymerase
8	*GT1*	9713..10870	34.63	Glycos_transf_1 family (PF00534.17) *E* value=1.4e-20	glycosyl transferase family protein [*Klebsiella oxytoca*] (SBM07213)	41/55(385)	Glycosyl transferase
9	*orf9*	10893..12008	36.11	Cellulase domain (PF00150.15) *E* value=4e-11	hypothetical protein [*Enterobacter aerogenes*] (WP_052320170)	99/99(371)	Hypothetical protein
10	*GT2*	12027..13109	33.98	Glycos_transf_1 family (PF00534.17) *E* value=2.3e-30	D-inositol-3-phosphate glycosyltransferase [*Serratia marcescens*] (CVG72219)	54/72(360)	D-inositol 3-phosphate glycosyltransferase
11	*manC*	13139..14560	41.42	NTP_transferase family (PF00483.20) *E* value=8.4e-75	mannose-1-phosphate guanylyltransferase/mannose-6-phosphate isomerase [*Shimwellia blattae*] (WP_002441831)	80/89(473)	Mannose-1-phosphate guanylyltransferase 1
12	*manB*	14577..15953	39.65	PGM_PMM_I domain (PF02878.13) *E* value=17.e-32	phosphomannomutase [*Enterobacter aerogenes*] (WP_042896015)	99/99(458)	Phosphomannomutase/phosphoglucomutase
13	*GT3*	16025..17179	35.32	Glycos_transf_1 family (PF00534.17) *E* value=2.8e-23	glycosyl transferase family 1 [*Enterobacter aerogenes*] (WP_042896017)	99/99(384)	GDP-mannose-dependent alpha-(1-6)-phosphatidylinositol monomannoside mannosyltransferase
14	*neuA*	17222..17905	39.04	CTP_transf_3 family (PF02348.16) *E* value=4.7e-30	CMP-N-acetylneuraminic acid synthetase [*Pluralibacter gergoviae*] (WP_048285306)	78/87(227)	N-acylneuraminate cytidylyltransferase
15	*neuB*	17916..20156	39.13	NeuB domain (PF03102.11)	acetylneuraminic acid synthetase	78/88(746)	N,N'-diacetyllegionaminic acid synthase

				E value=9.1e-84	[*Klebsiella pneumoniae*] (WP_062794592)		
16	*wzx*	20156..21406	34.21	Polysacc_synt family (PF01943.14) *E* value=1.9e-11	Wzx [*Shigella dysenteriae*] (ACD37093)	27/52(416)	Flippase
17	*AT*	21396..21902	34.71	Hexapep repeat (PF00132.21) *E* value=3.1e-10	acetyltransferase [*Serratia plymuthica*] (WP_041416845)	64/76(168)	Putative acetyltransferase
18	*orf18*	21965..22921	32.71		hypothetical protein [*Enterobacter aerogenes*] (WP_052320171)	97/98(318)	Hypothetical protein

E. aerogenes PSgc7							
Orf no.	Gene name	Position of gene	G+C content (%)	Conserved domain(s)	Similar protein(s), strain(s) (Genbank accession No.)	%Identical /%Similar (total No. of aa)	Putative function of protein
1	*cpsACP*	1..630	51.43	PAP2 superfamily (PF01569.18) *E* value=4.6e-09	acid phosphatase, [*Klebsiella oxytoca*] (SAQ01243)	73/84 (209)	Acid phosphatase
2	*wzi*	1563..2996	49.72	Caps_assemb_Wzi family (PF14052.3) *E* value= 1.6e-126	surface assembly of capsule [*Klebsiella pneumoniae*] (BAO24050)	88/93 (477)	Capsule assembly protein Wzi
3	*wza*	3119..4258	40.35	Polysaccharide biosynthesis/export protein family (PF02563.13) *E* value= 3.4e-28	polysaccharide export protein, [*Klebsiella pneumoniae*] (WP_032441376)	92/96 (379)	Polysaccharide biosynthesis/export protein
4	*wzb*	4258..4695	42.92	LMWPc domain (PF01451.18) *E* value= 1.4e-40	protein tyrosine phosphatase [*Klebsiella oxytoca*] (AEX06753）	73/81 (145)	Low molecular weight protein-tyrosine-phosphatase Wzb
5	*wzc*	4710..6872	37.82	Wzz family (PF02706.12) *E* value=1.4e-46	Tyrosine-protein kinase Wzc, [*Enterobacter cloacae*] (SAJ11239.1)	99/99 (720)	Putative tyrosine-protein kinase in cps region
6	*wbaP*	6979..8403	37.54	Bac_transf family (PF02397.13) *E* value=1.6e-57	undecaprenyl-phosphate galactose phosphotransferase WbaP [*Enterobacter aerogenes*] (WP_026612258)	100/100(474)	Undecaprenyl-phosphate galact[illegible] phosphotransferase

7	*GT1*	8439..9347	36.63	Glycos_transf_2 family (PF00535.23) *E* value=3.6e-18	rhamnosyltransferase [*Enterobacter aerogenes*] (WP_059304719)	99/100(302)	N-glycosyltransferase
8	*GT2*	9494..10351	35.9	Glycos_transf_2 family (PF00535.23) *E* value=2.4e-32	glycosyl transferase family 2 [*Enterobacter aerogenes*] (WP_032708707)	63/79(285)	GalNAc(5)-diNAcBac-PP-undecaprenol beta-1,3-glucosyltransferase
9	*GT3*	10381..11220	35.12	Glycos_transf_2 family (PF00535.23) *E* value=4e-7	glycosyl transferase family 2 [*Enterobacter aerogenes*] (WP_047053237)	99/100(279)	N-acetylglucosaminyl-diphospho-decaprenol L-rhamnosyltransferase
10	*orf10*	11234..12748	35.58		hypothetical protein [*Serratia rubidaea*] (WP_054304887)	62/76(504)	Hypothetical protein
11	*wzy*	12848..13999	35.42		Lipid A core-O-antigen ligase and related enzymes [*Klebsiella oxytoca*] (SBL45261)	28/49(383)	Polymerase

E. aerogenes PSgc8							
Orf no.	Gene name	Position of gene	G+C content (%)	Conserved domain(s)	Similar protein(s), strain(s) (Genbank accession No.)	%Identical /%Similar (total No. of aa)	Putative function of protein
1	*cpsACP*	1..630	51.59	PAP2 superfamily (PF01569.18) *E* value=4.6e-09	acid phosphatase, [*Klebsiella oxytoca*] (SAQ01243)	73/84 (209)	Acid phosphatase
2	*wzi*	1563..2996	49.02	Caps_assemb_Wzi family (PF14052.3) *E* value= 1.6e-126	surface assembly of capsule [*Klebsiella pneumoniae*] (BAO24050)	88/93 (477)	Capsule assembly protein Wzi
3	*wza*	3108..4244	42.57	Polysaccharide biosynthesis/export protein family (PF02563.13) *E* value= 3.4e-28	polysaccharide export protein, [*Klebsiella pneumoniae*] (WP_032441376)	92/96 (378)	Polysaccharide biosynthesis/export protein
4	*wzb*	4247..4684	40.41	LMWPc domain (PF01451.18) *E* value= 1.4e-40	protein tyrosine phosphatase [*Klebsiella oxytoca*] (AEX06753)	73/81 (145)	Low molecular weight protein-tyrosine-phosphatase Wzb
5	*wzc*	4699..6864	37.67	Wzz family (PF02706.12) *E* value=1.4e-46	Tyrosine-protein kinase Wzc, [*Enterobacter cloacae*] (SAJ11239.1)	99/99 (720)	Putative tyrosine-protein kinase in cps region
6	*GT1*	6968..7957	30.61	Glycos_transf_2 family (PF00535.23) *E* value=3.3e-24	glycosyl transferase [*Klebsiella sp*. 2212/52] (BAT24112)	56/78(291)	UDP-Glc:alpha-D-GlcNAc-diphosphoundecapre beta-1,3-glucosyltransferase WfaP
7	*GT2*	7967..8800	35.37	Glycos_transf_2	glycosyl transferase [*Klebsiella*	53/68(277)	Putative glycosyl transferase

				family (PF00535.23) *E* value=2.5e-18	*sp*. 2212/52] (BAT24111)		
8	*wzy*	8840..10096	31.98	EpsG family (PF14897.3) *E* value=1.2e-17	O-antigen and lipid-linked capsular repeat unit polymerase [*Klebsiella sp*. SW4] (BAT24203)	61/80(418)	Polymerase
9	*orf9*	10143..11171	32.75	Glyco_hydro_cc family (PF11790.5) *E* value=1.2e-4	hypothetical protein [*Enterobacter aerogenes*] (WP_047036453)	98/99(342)	Hypothetical protein
10	*GT3*	11221..12210	34.04	Glyco_transf_4 family (PF13439.3) *E* value=4.6e-8	glycosyl transferase family 1 [*Enterobacter aerogenes*] (WP_045389163)	64/81(329)	D-inositol 3-phosphate glycosyltransferase
11	*GT4*	12349..13503	37.49	Glycos_transf_1 family (PF00534.17) *E* value=4e-26	glycosyl transferase family 1 [*Enterobacter aerogenes*] (WP_047042011)	78/90(384)	GDP-mannose-dependent alpha-(1-6)-phosphatidylinositol monomannos mannosyltransferase
12	*wcaJ*	13513..14976	36.75	Bac_transf family (PF02397.13) *E* value=2.1e-57	undecaprenyl-phosphate glucose phosphotransferase [*Enterobacter aerogenes*] (WP_048229034)	100/100(487)	UDP-glucose:undecaprenyl-phosphate glucose-1-phosphate transferase
13	*wzx*	14985..16352	33.77	Polysacc_synt family (PF01943.14) *E* value=2.2e-25	flippase [*Klebsiella sp*. SW4] (BAT24209)	67/85(455)	Flippase
14	*PT*	16358..17518	34.45	PS_pyruv_trans family (PF04230.10) *E* value=1.6e-21	putative pyruvyl transferase *[Klebsiella sp*. 708] (BAT24411)	53/72(386)	Polysaccharide pyruvyl transferase
15	*GT5*	17554..18393	31.07		glycosyl transferase [*Serratia*	45/65(279)	Glycosyl transferase

	plymuthica S13] (AGP46915)	

E. aerogenes PSgc9							
Orf no.	Gene name	Position of gene	G+C content (%)	Conserved domain(s)	Similar protein(s), strain(s) (Genbank accession No.)	%Identical /%Similar (total No. of aa)	Putative function of protein
1	*cpsACP*	1..630	50.32	PAP2 superfamily (PF01569.18) *E* value=4.6e-09	acid phosphatase, [*Klebsiella oxytoca*] (SAQ01243)	73/84 (209)	Acid phosphatase
2	*wzi*	1565..3004	49.72	Caps_assemb_Wzi family (PF14052.3) *E* value= 1.6e-126	surface assembly of capsule [*Klebsiella pneumoniae*] (BAO24050)	88/93 (479)	Capsule assembly protein Wzi
3	*wza*	3115..4251	42.3	Polysaccharide biosynthesis/export protein family (PF02563.13) *E* value= 3.4e-28	polysaccharide export protein, [*Klebsiella pneumoniae*] (WP_032441376)	92/96 (378)	Polysaccharide biosynthesis/export protein
4	*wzb*	4254..4688	42.07	LMWPc domain (PF01451.18) *E* value= 1.4e-40	protein tyrosine phosphatase [*Klebsiella oxytoca*] (AEX06753)	73/81 (144)	Low molecular weight protein-tyrosine-phosphatase Wzb
5	*wzc*	4705..6858	37.14	Wzz family (PF02706.12) *E* value=1.4e-46	Tyrosine-protein kinase Wzc, [*Enterobacter cloacae*] (SAJ11239.1)	99/99 (717)	Putative tyrosine-protein kinase in cps region
6	*wbaP*	6932..8380	37.13	Bac_transf family (PF02397.13) *E* value=7.2e-58	undecaprenyl-phosphate galactose phosphotransferase WbaP [*Enterobacter aerogenes*]	97/98(482)	undecaprenyl-phosphate galactose phosphotransferase

					(WP_047046471)		
7	*orf7*	8401..9765	29.6	NosD domain (PF05048.10) *E* value=7.6e-7	hypothetical protein [*Enterobacter aerogenes*] (WP_047046473)	89/95(454)	
8	*wzy*	9803..11029	33.74		Lipid A core-O-antigen ligase [*Leisingera aquaemixtae*] (CUI01125)	29/50(408)	Polymerase
9	*GT1*	11022..12068	32.57	Glycos_transf_1 family (PF00534.17) *E* value=2.4e-20	group 1 glycosyl transferase [*Serratia plymuthica*] (WP_006320961)	40/60(348)	UDP-D-galactose:(glucosyl)lipopolysaccharide-1, 6-D-galactosyltransferase
10	*GT2*	12082..13065	36.69	Glycos_transf_1 family (PF00534.17) *E* value=2.2e-29	glycosyl transferase family 1 [*Enterobacter aerogenes*] (WP_047078095)	97/99(327)	Alpha-D-kanosaminyltransferase
11	*wzx*	13127..14668	34.44		flippase [*Klebsiella sp*. 7444] (BAT23657)	48/68(513)	Flippase
12	*PT*	14670..15659	33.94	PS_pyruv_tran family (PF04230.10) *E* value=3.4e-14	putative pyruvyl transferase [*Klebsiella sp*. 6168] (BAT23618)	64/80(329)	Polysaccharide pyruvyl transferase
13	*manC*	15715..17133	41.16	NTP_transferase family (PF00483.20) *E* value=3.6e-74	mannose-1-phosphate guanyltransferase [*Enterobacter aerogenes*] (WP_049056265)	100/100(472)	Mannose-1-phosphate guanylyltransferase 1
14	*manB*	17150..18523	40.68	PGM_PMM_I domain (PF02878.13) *E* value=1.2e-32	phosphomannomutase [*Enterobacter aerogenes*] (WP_049047037)	100/100(457)	Phosphomannomutase/phosphoglucomutase
15	*GT3*	18607..19758	37.41	Glycos_transf_1	Glycosyltransferase [*Klebsiella*	81/90(383)	GDP-mannose-dependent

	family (PF00534.17)	*variicola*]		alpha-(1-6)-phosphatidylinositol
	E value=3.3e-28	(CTQ06038)		monomannoside mannosyltransferase

E. aerogenes PSgc10							
Orf no.	Gene name	Position of gene	G+C content (%)	Conserved domain(s)	Similar protein(s), strain(s) (Genbank accession No.)	%Identical /%Similar (total No. of aa)	Putative function of protein
1	*cpsACP*	1..630	51.11	PAP2 superfamily (PF01569.18) *E* value=4.6e-09	acid phosphatase, [*Klebsiella oxytoca*] (SAQ01243)	73/84 (209)	Acid phosphatase
2	*wzi*	1563..3002	48.47	Caps_assemb_Wzi family (PF14052.3) *E* value= 1.6e-126	surface assembly of capsule [*Klebsiella pneumoniae*] (BAO24050)	88/93 (479)	Capsule assembly protein Wzi
3	*wza*	3106..4242	43.54	Polysaccharide biosynthesis/export protein family (PF02563.13) *E* value= 3.4e-28	polysaccharide export protein, [*Klebsiella pneumoniae*] (WP_032441376)	92/96 (378)	Polysaccharide biosynthesis/export protein
4	*wzb*	4245..4679	43.22	LMWPc domain (PF01451.18) *E* value= 1.4e-40	protein tyrosine phosphatase [*Klebsiella oxytoca*] (AEX06753)	73/81 (144)	Low molecular weight protein-tyrosine-phosphatase Wzb
5	*wzc*	4697..6856	38.8	Wzz family (PF02706.12) *E* value=1.4e-46	Tyrosine-protein kinase Wzc, [*Enterobacter cloacae*] (SAJ11239.1)	99/99 (719)	Putative tyrosine-protein kinase in cps region
6	*wbaP*	6967..8394	37.04	Bac_transf family (PF02397.13) *E* value=2.9e-57	undecaprenyl-phosphate galactose phosphotransferase WbaP [*Enterobacter aerogenes*] (WP_032707143)	100/100(475)	Undecaprenyl-phosphate galactose phosphotransferase

7	*wzx*	8460..9905	34.16	Polysacc_synt_3 family (PF13440.3) *E* value=1.9e-88	flippase [*Raoultella planticola*] (BAF47073)	53/75(481)	Flippase
8	*PT*	9905..10969	31.17	PS_pyruv_trans family (PF04230.10) *E* value=3e-26	pyruvyl transferase [*Klebsiella sp*. 265(1)] (BAT24268)	76/87(354)	Polysaccharide pyruvyl transferase
9	*GT1*	11008..12156	32.11	Glycos_transf_1 family (PF00534.17) *E* value=5.2e-32	group 1 glycosyl transferase [*Klebsiella oxytoca*] (SAQ07483)	76/88(382)	GDP-mannose-dependent alpha-(1-6)-phosphatidylinositol monomannoside mannosyltransferase
10	*GT2*	12131..13252	35.83	Glycos_transf_1 family (PF00534.17) *E* value=4e-27	glycosyl transferase, group 1 family [*Klebsiella pneumoniae*] (BAF75759)	59/76(373)	Glycosyl transferase, group 1
11	*wzy*	13361..14602	34.38		O-antigen polymerase [*Klebsiella sp*. 265(1)] (BAT24271)	87/95(413)	Polymerase
12	*manC*	14642..16060	38.05	NTP_transferase family (PF00483.20) *E* value=1.1e-70	mannose-1-phosphate guanyltransferase [*Enterobacter aerogenes*] (KJF84403)	99/100(472)	Mannose-1-phosphate guanylyltransferase 1
13	*manB*	16076..17452	38.85	PGM_PMM_I domain (PF02878.13) *E* value=6e-32	phosphomannomutase [*Enterobacter aerogenes*] (WP_032706030)	100/100(458)	Phosphomannomutase/phosphoglucomutase
14	*GT3*	17516..18658	35.52	Glycos_transf_1 family (PF00534.17) *E* value=3.4e-27	glycosyl transferase family 1 [*Klebsiella oxytoca*] (WP_042945668)	88/93(380)	GDP-mannose-dependent alpha-(1-6)-phosphatidylinositol monomannoside mannosyltransferase
15	*orf15*	18730..19818	35.45	Cellulase domain	hypothetical protein	98/99(362)	Funtion unknown

	(PF00150.15)	[*Enterobacter aerogenes*]
	E value=3.5e-13	(WP_047038707)

E. aerogenes PSgc11							
Orf no.	Gene name	Position of gene	G+C content (%)	Conserved domain(s)	Similar protein(s), strain(s) (Genbank accession No.)	%Identical /%Similar (total No. of aa)	Putative function of protein
1	*cpsACP*	1..630	49.52	PAP2 superfamily (PF01569.18) *E* value=4.6e-09	acid phosphatase, [*Klebsiella oxytoca*] (SAQ01243)	73/84 (209)	Acid phosphatase
2	*wzi*	1552..2991	48.47	Caps_assemb_Wzi family (PF14052.3) *E* value= 1.6e-126	surface assembly of capsule [*Klebsiella pneumoniae*] (BAO24050)	88/93 (479)	Capsule assembly protein Wzi
3	*wza*	3102..4238	44.59	Polysaccharide biosynthesis/export protein family (PF02563.13) *E* value= 3.4e-28	polysaccharide export protein, [*Klebsiella pneumoniae*] (WP_032441376)	92/96 (378)	Polysaccharide biosynthesis/export protein
4	*wzb*	4240..4674	42.53	LMWPc domain (PF01451.18) *E* value= 1.4e-40	protein tyrosine phosphatase [*Klebsiella oxytoca*] (AEX06753)	73/81 (144)	Low molecular weight protein-tyrosine-phosphatase Wzb
5	*wzc*	4691..6856	38.78	Wzz family (PF02706.12) *E* value=1.4e-46	Tyrosine-protein kinase Wzc, [*Enterobacter cloacae*] (SAJ11239.1)	99/99 (721)	Putative tyrosine-protein kinase in cps region
6	*wbaP*	6958..8385	37.04	Bac_transf family (PF02397.13) *E* value=2.5e-56	UDP-phosphate galactose phosphotransferase [*Enterobacter aerogenes*] (WP_049047047)	98/98(475)	Undecaprenyl-phosphate galactose phosphotransferase

7	*GT1*	8498..9544	34.48	Glycos_transf_1 family (PF00534.17) *E* value=1.1e-18	group 1 glycosyl transferase [*Serratia plymuthica*] (WP_006320961)	40/58(378)	UDP-D-galactose:(glucosyl)lipopolysaccharide-1 6-D-galactosyltransferase
8	*GT2*	9562..10545	37.4	Glycos_transf_1 family (PF00534.17) *E* value=1.2e-29	glycosyl transferase family 1 [*Enterobacter aerogenes*] (WP_049061328)	100/100(327)	Alpha-D-kanosaminyltransferase
9	*wzx*	10607..12148	35.41		flippase [*Klebsiella sp*. 7444] (BAT23657)	48/68(513)	Flippase
10	*PT*	12150..13139	35.25	PS_pyruv_trans family (PF04230.10) *E* value=6.3e-14	putative pyruvyl transferase [*Klebsiella sp*. 7444] (BAT23658)	63/79(329)	Polysaccharide pyruvyl transferase
11	*manC*	13195..14616	40.51	NTP_transferase family (PF00483.20) *E* value=6.9e-74	mannose-1-phosphate guanyltransferase [*Enterobacter aerogenes*] (WP_049047039)	97/98(473)	Mannose-1-phosphate guanylyltransferase 1
12	*manB*	14633..16006	41.05	PGM_PMM_I domain (PF02878.13) *E* value=1.2e-32	phosphomannomutase [*Enterobacter aerogenes*] (WP_049061326)	100/100(457)	Phosphomannomutase/phosphoglucomutase
13	*GT3*	16089..17240	37.76	Glyco_transf_4 domain (PF13439.3) *E* value=4.2e-12	Glycosyltransferase [*Klebsiella variicola*] (CTQ06038)	82/89(383)	GDP-mannose-dependent alpha-(1-6)-phosphatidylinositol monomannos mannosyltransferase

E. aerogenes PSgc12							
Orf no.	Gene name	Position of gene	G+C content (%)	Conserved domain(s)	Similar protein(s), strain(s) (Genbank accession No.)	%Identical /%Similar (total No. of aa)	Putative function of protein
1	*cpsACP*	1..630	51.43	PAP2 superfamily (PF01569.18) *E* value=4.6e-09	acid phosphatase, [*Klebsiella oxytoca*] (SAQ01243)	73/84 (209)	Acid phosphatase
2	*wzi*	1563..2996	48.81	Caps_assemb_Wzi family (PF14052.3) *E* value= 1.6e-126	surface assembly of capsule [*Klebsiella pneumoniae*] (BAO24050)	88/93 (477)	Capsule assembly protein Wzi
3	*wza*	3108..4244	43.1	Polysaccharide biosynthesis/export protein family (PF02563.13) *E* value= 3.4e-28	polysaccharide export protein, [*Klebsiella pneumoniae*] (WP_032441376)	92/96 (378)	Polysaccharide biosynthesis/export protein
4	*wzb*	4247..4717	39.49	LMWPc domain (PF01451.18) *E* value= 1.4e-40	protein tyrosine phosphatase [*Klebsiella oxytoca*] (AEX06753)	73/81 (156)	Low molecular weight protein-tyrosine-phosphatase Wzb
5	*wzc*	4698..6863	36.61	Wzz family (PF02706.12) *E* value=1.4e-46	Tyrosine-protein kinase Wzc, [*Enterobacter cloacae*] (SAJ11239.1)	99/99 (721)	Putative tyrosine-protein kinase in cps region
6	*wzy*	7086..8342	31.19	EpsG family (PF14897.3) *E* value=7.3e-19	O-antigen and lipid-linked capsular repeat unit polymerase [*Klebsiella sp.* 1702] (BAT23784)	41/60(418)	Polymerase

7	*orf7*	8398..9423	32.65	Cellulase family (PF00150.15) *E* value=1.5e-11	hypothetical protein SR67_19405 [*Enterobacter aerogenes*] (KJP10107)	99/100(341)	Function unknown
8	*GT1*	9520..10512	33.94	Glycos_transf_1 family (PF00534.17) *E* value=9.7e-29	glycosyl transferase [*Klebsiella sp*. SW4] (BAT24205)	75/87(330)	D-inositol 3-phosphate glycosyltransferase
9	*GT2*	10616..11725	29.91	Glyco_trans_1_4 domain (PF13692.3) *E* value=2.2e-6	glycosyl transferase [*Klebsiella sp*. SW4] (BAT24206)	63/81(369)	Putative teichuronic acid biosynthe glycosyltransferase TuaH
10	*GT3*	11877..13031	35.76	Glyco_transf_4 domain (PF13439.3) *E* value=2.8e-11	glycosyl transferase family 1 [*Klebsiella pneumoniae*] (WP_032733510)	79/90(384)	GDP-mannose-dependent alpha-(1-6)-phosphatidylinositol monomannos mannosyltransferase
11	*wcaJ*	13101..14522	36.36	Bac_transf family (PF02397.13) *E* value=1e-56	undecaprenyl-phosphate glucose phosphotransferase [*Klebsiella sp. SW4*] (BAT24208)	90/95(473)	UDP-glucose:undecaprenyl-phosphate glucose-1-phosphate transferase
12	*wzx*	14584..15903	35.83	Polysacc_synt family (PF01943.14) *E* value=2.1e-22	flippase [*Escherichia coli*] (WP_001581854)	63/80(439)	Flippase
13	*PT*	15905..17056	32.99	PS_pyruv_trans family (PF04230.10) *E* value=5.7e-27	pyruvyl transferase [*Klebsiella sp*. SW4] (BAT24210)	80/90(383)	Polysaccharide pyruvyl transferase
14	*GT4*	17092..17937	33.69		glycosyl transferase [*Klebsiella sp*. SW4] (BAT24211)	62/75(281)	Glycosyltransferase

E. aerogenes PSgc13							
Orf no.	Gene name	Position of gene	G+C content (%)	Conserved domain(s)	Similar protein(s), strain(s) (Genbank accession No.)	%Identical /%Similar (total No. of aa)	Putative function of protein
1	*cpsACP*	1..630	51.27	PAP2 superfamily (PF01569.18) *E* value=4.6e-09	acid phosphatase, [*Klebsiella oxytoca*] (SAQ01243)	73/84 (209)	Acid phosphatase
2	*wzi*	1563..2996	48.61	Caps_assemb_Wzi family (PF14052.3) *E* value= 1.6e-126	surface assembly of capsule [*Klebsiella pneumoniae*] (BAO24050)	88/93 (477)	Capsule assembly protein Wzi
3	*wza*	3108..4244	43.71	Polysaccharide biosynthesis/export protein family (PF02563.13) *E* value= 3.4e-28	polysaccharide export protein, [*Klebsiella pneumoniae*] (WP_032441376)	92/96 (378)	Polysaccharide biosynthesis/export protein
4	*wzb*	4247..4681	38.39	LMWPc domain (PF01451.18) *E* value= 1.4e-40	protein tyrosine phosphatase [*Klebsiella oxytoca*] (AEX06753)	73/81 (144)	Low molecular wei protein-tyrosine-phosphatase Wzb
5	*wzc*	4697..6862	35.73	Wzz family (PF02706.12) *E* value=1.4e-46	Tyrosine-protein kinase Wzc, [*Enterobacter cloacae*] (SAJ11239.1)	99/99 (721)	Putative tyrosine-protein kinase in cps region
6	*wbaP*	6961..8385	35.65	Bac_transf family (PF02397.13) *E* value=2.3e-57	undecaprenyl-phosphate galactose phosphotransferase WbaP [*Enterobacter aerogenes*] (WP_046883244)	99/100(474)	undecaprenyl-phosphate galacto phosphotransferase

7	*GT1*	8398..9561	32.04	Glycos_transf_1 family (PF00534.17) *E* value=1.9e-21	glycosyl transferase family 1 [*Enterobacter aerogenes*] (WP_045361541)	100/100(387)	
8	*GT2*	9548..10555	33.53	Glyco_transf_4 domain (PF13439.3) *E* value=4.9e-8	glycosyl transferase family 1 [*Klebsiella pneumoniae*] (WP_049186716)	99/99(335)	N-acetylgalactosamine-N, N'-diacetylbacillosaminyl-diphospho-undecapren 4-alpha-N-acetylgalactosaminyltransferase
9	*GT3*	10601..11740	36.84	Glycos_transf_1 family (PF00534.17) *E* value=6.5e-27	glycosyl transferase family 1 [*Raoultella ornithinolytica*] (WP_041147694)	73/85(379)	GDP-mannose-dependent alpha-(1-6)-phosphatidylinositol monomannos mannosyltransferase
10	*wzy*	11807..12943	34.21	Wzy_C family (PF04932.12) *E* value=3.2e-6		(378)	Polymerase
11	*GH*	13231..13983	35.86		putative glycosyl hydrolase [*Klebsiella sp.* 7444] (dbj|BAT23653.1|)	45/61(250)	
12	*GT4*	14016..15233	33.09		colanic acid biosynthesis glycosyltransferase WcaL [*Rahnella aquatilis*] (ref|WP_047605896.1|)	55/73(405)	GDP-mannose-dependent alpha-(1-6)-phosphatidylinositol monomannos mannosyltransferase
13	*PT*	15247..16263	37.17		Polysaccharide pyruvyl transferase [*Klebsiella pneumoniae*] (CTQ28985)	58/74(338)	Exopolysaccharide gluco ketal-pyruvate-transferase
14	*wzx*	16314..17747	34.59	Polysacc_synt_C family (PF14667.3) *E* value=2.6e-13	lipopolysaccharide biosynthesis protein [*Rahnella aquatilis*] (WP_015698542)	66/79(477)	Flippase

15	*GT5*	17749..18741	31.42	Glycos_transf_2 family (PF00535.23) *E* value=1.8e-31	glycosyl transferase [*Rahnella aquatilis*] (WP_047610754)	47/67(330)	Putative glycosyltransferase EpsH

E. aerogenes PSgc14							
Orf no.	Gene name	Position of gene	G+C content (%)	Conserved domain(s)	Similar protein(s), strain(s) (Genbank accession No.)	%Identical /%Similar (total No. of aa)	Putative function of protein
1	*cpsACP*	1..630		PAP2 superfamily (PF01569.18) *E* value=4.6e-09	acid phosphatase, [*Klebsiella oxytoca*] (SAQ01243)	73/84 (209)	Acid phosphatase
2	*wzi*	1563..3002		Caps_assemb_Wzi family (PF14052.3) *E* value= 1.6e-126	surface assembly of capsule [*Klebsiella pneumoniae*] (BAO24050)	88/93 ()	Capsule assembly protein Wzi
3	*wza*	3106..4242		Polysaccharide biosynthesis/export protein family (PF02563.13) *E* value= 3.4e-28	polysaccharide export protein, [*Klebsiella pneumoniae*] (WP_032441376)	92/96 ()	Polysaccharide biosynthesis/export protein
4	*wzb*	4245..4679		LMWPc domain (PF01451.18) *E* value= 1.4e-40	protein tyrosine phosphatase [*Klebsiella oxytoca*] (AEX06753)	73/81 ()	Low molecular weight protein-tyrosine-phosphatase Wzb
5	*wzc*	4697..6856		Wzz family (PF02706.12) *E* value=1.4e-46	Tyrosine-protein kinase Wzc, [*Enterobacter cloacae*] (SAJ11239.1)	99/99 ()	Putative tyrosine-protein kinase in cps region
6	*wbaP*	6967..8349		Bac_transf family (PF02397.13) *E* value=3.4e-53			undecaprenyl-phosphate galacto phosphotransferase
7	*manB*	8818..9765		PGM_PMM_II			Phosphomannomutase/phosphoglucomutase

			domain (PF02879.13)<br>*E* value=2.6e-24	
8	*ugd*	9982..10890	UDPG_MGDP_dh<br>domain (PF00984.16)<br>*E* value=4.8e-26	UDP-glucose 6-dehydrogenase

E. aerogenes PSgc15							
Orf no.	Gene name	Position of gene	G+C content (%)	Conserved domain(s)	Similar protein(s), strain(s) (Genbank accession No.)	%Identical /%Similar (total No. of aa)	Putative function of protein
1	*cpsACP*	1..630		PAP2 superfamily (PF01569.18) *E* value=4.6e-09	acid phosphatase, [*Klebsiella oxytoca*] (SAQ01243)	73/84 (209)	Acid phosphatase
2	*wzi*	1561..2994		Caps_assemb_Wzi family (PF14052.3) *E* value= 1.6e-126	surface assembly of capsule [*Klebsiella pneumoniae*] (BAO24050)	88/93 ()	Capsule assembly protein Wzi
3	*wza*	3115..4251		Polysaccharide biosynthesis/export protein family (PF02563.13) *E* value= 3.4e-28	polysaccharide export protein, [*Klebsiella pneumoniae*] (WP_032441376)	92/96 ()	Polysaccharide biosynthesis/export protein
4	*wzb*	4242..4688		LMWPc domain (PF01451.18) *E* value= 1.4e-40	protein tyrosine phosphatase [*Klebsiella oxytoca*] (AEX06753)	73/81 ()	Low molecular weight protein-tyrosine-phosphatase Wzb
5	*wzc*	4706..6868		Wzz family (PF02706.12) *E* value=1.4e-46	Tyrosine-protein kinase Wzc, [*Enterobacter cloacae*] (SAJ11239.1)	99/99 ()	Putative tyrosine-protein kinase in cps region
6	*wbaP*	6965..8404		Bac_transf family (PF02397.13) *E* value=9.5e-58			undecaprenyl-phosphate galacto phosphotransferase
7	*IS*	8632..9135		DDE_Tnp_IS1 family			IS1 transposase

			(PF03400.10)<br>*E* value=9.2e-75		
8	*GT1*	9211..10122	Glycos_transf_2 family (PF00535.23)<br>*E* value=6.7e-14		Glycosyl transferase
9	*GT2*	10238..11104	Glycos_transf_2 family (PF00535.23)<br>*E* value=5.6e-29		GalNAc(5)-diNAcBac-PP-undecaprenol beta-1,3-glucosyltransferase
10	*GT3*	11129..11968	Glycos_transf_2 family (PF00535.23)<br>*E* value=6.4e-7		Glycosyl transferase family 2
