# Supplementary material for: Establishment of a Molecular Serotyping Scheme and a Multiplexed Luminex-Based Array for Enterobacter aerogenes
Source: Front Microbiol. 2018 Mar 19;9:501. doi: 10.3389/fmicb.2018.00501 (PMC5867348; doi:10.3389/fmicb.2018.00501)
Supplement: Supplementary file 4 [file Image_1.PDF]

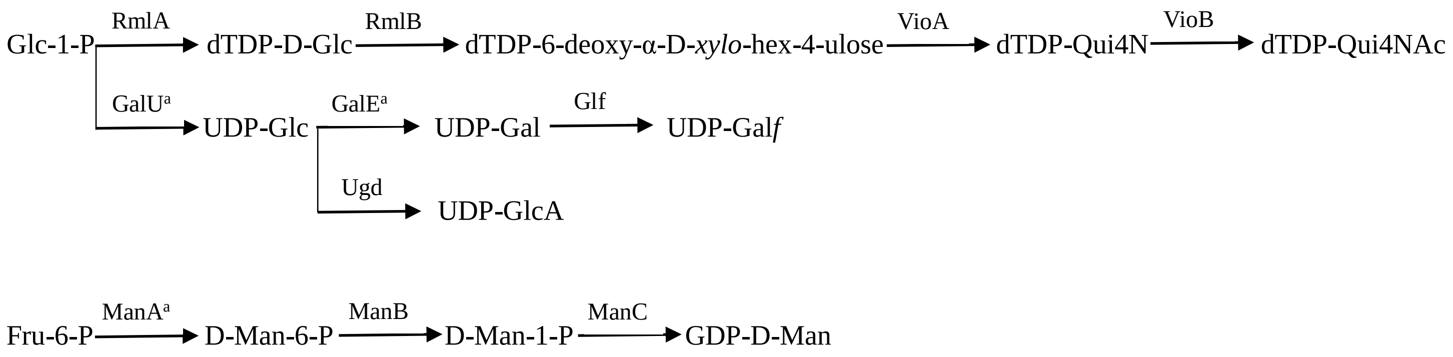

Supplementary figure 1. Biosynthetic pathways for putative rare sugars in *E. aerogenes* surface polysaccharide. RmlA, glucose-1-phosphate thymidylyltransferase; RmlB, dTDP-D-glucose 4,6-dehydratase; VioA, aminotransferase; VioB, acetyltransferase; GalU, UTP-glucose-1-phosphate uridylyltransferase; GalE, UDP-glucose-4-epimerase; Glf, UDP-galactopyranose mutase; Ugd, UDP-glucose 6-dehydrogenase; ManA, phosphomannose isomerase; ManB, phosphomannomutase; ManC, mannose-1-phosphate guanylyltransferase. [a]The enzymes is encoded by the genes located outside the PSgc.
