# Supplementary material for: Establishment of a Molecular Serotyping Scheme and a Multiplexed Luminex-Based Array for Enterobacter aerogenes
Source: Front Microbiol. 2018 Mar 19;9:501. doi: 10.3389/fmicb.2018.00501 (PMC5867348; doi:10.3389/fmicb.2018.00501)
Supplement: Supplementary file 5 [file Image_2.PDF]

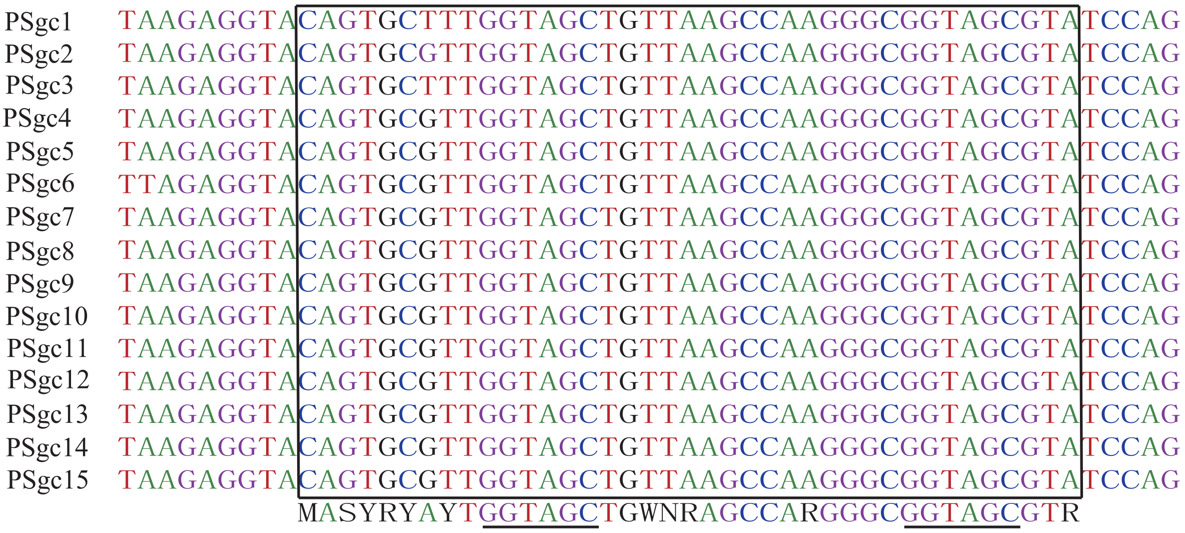

Supplementary figure 2. JUMPstar sequences between *cpsACP* and *wzi* genes of all PSgc types. The 39 bp JUMPstar sequence is boxed, and a consensus sequence (20) is shown undrneath. A repeated 6 bp motif within the consensus is underlined. M, A or C; S, G or C; Y, C or T; R, G or A; W, T or A; N, T or G or C or A.
