# Supplementary material for: Establishment of a Molecular Serotyping Scheme and a Multiplexed Luminex-Based Array for Enterobacter aerogenes
Source: Front Microbiol. 2018 Mar 19;9:501. doi: 10.3389/fmicb.2018.00501 (PMC5867348; doi:10.3389/fmicb.2018.00501)
Supplement: Supplementary file 7 [file Image_4.PDF]

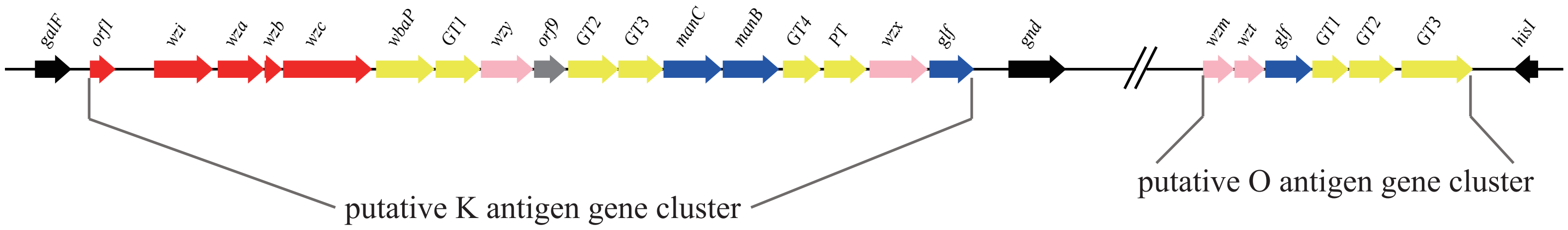

Supplementary figure 4. Schematic diagram of putative capsular antigen gene cluster and O antigen gene cluster of *E. aerogenes* CAV1320
